# Blockade of the forward Na^+^/Ca^2+^ exchanger suppresses the growth of glioblastoma cells through Ca^2+^‐mediated cell death

**DOI:** 10.1111/bph.14692

**Published:** 2019-06-17

**Authors:** Hui‐Jie Hu, Shan‐Shan Wang, Yan‐Xia Wang, Yan Liu, Xue‐Mei Feng, Ying Shen, Liang Zhu, Hong‐Zhuan Chen, Mingke Song

**Affiliations:** ^1^ Department of Pharmacology and Chemical Biology, Institute of Medical Sciences Shanghai Jiao Tong University School of Medicine Shanghai China

## Abstract

**Background and Purpose:**

The Na^+^/Ca^2+^ exchanger (NCX) working in either forward or reverse mode participates in maintaining intracellular Ca^2+^ ([Ca^2+^]_i_) homeostasis, which is essential for determining cell fate. Previously, numerous blockers targeting reverse or forward NCX have been developed and studied in ischaemic tissue injury but barely examined in glioblastoma for the purpose of anti‐tumour therapy. We assessed the effect of NCX blockers on glioblastoma growth and whether NCX can become a therapeutic target.

**Experimental Approach:**

Patch‐clamp recording, Ca^2+^ imaging, flow cytometry, and Western blot were used to study the effects of specific and non‐specific NCX blockers on cultured glioblastoma cells. In vivo bioluminescent imaging was used to measure effects on grafted glioblastoma.

**Key Results:**

Selectively blocking the reverse NCX with SEA0400, SN‐6, and YM‐244769 did not affect tumour cell viability. Blocking the forward NCX with bepridil, CB‐DMB, or KB‐R7943 elevated [Ca^2+^]_i_ and killed glioblastoma cells. Bepridil and CB‐DMB caused Ca^2+^‐dependent cell cycle arrest together with apoptosis, which were all attenuated by a Ca^2+^ chelator BAPTA‐AM. Systemic administration of bepridil inhibited growth of brain‐grafted glioblastoma. Bepridil did not appear to have a cytotoxic effect on human astrocytes, which have higher functional expression of NCX than glioblastoma cells.

**Conclusions and Implications:**

Low expression of the NCX makes glioblastoma cells sensitive to disturbance of [Ca^2+^]_i_. Interventions designed to block the forward NCX can cause Ca^2+^‐mediated injury to glioblastoma thus having therapeutic potential. Bepridil could be a lead compound for developing new anti‐tumour drugs.

**Abbreviations**
[Ca^2+^]_i_: intracellular Ca^2+^
BAPTA‐AM: 1,2‐bis(2‐aminophenoxy)ethane‐*N*,*N*,*N*′,*N*′‐tetraacetic acid tetrakis (acetoxymethyl ester)
BBB: blood–brain barrier
bepridil: *N*‐benzyl‐*N*‐(3‐isobutoxy‐2‐pyrrolidin‐1‐yl‐propyl)aniline
CB‐DMB: [*N*‐(4‐chlorobenzyl)]2,4‐dimethylbenzamyl
CC‐930: tanzisertib
KB‐R7943: 2‐[4‐[(4‐nitrophenyl)methoxy]phenyl]ethyl ester carbamimidothioic acid methanesulfonate
NCX: Na^+^/Ca^2+^ exchanger
SEA0400: 2‐[4‐[(2,5‐difluorophenyl)methoxy]phenoxy]‐5‐ethoxyaniline
SN‐6: 2‐[[4‐[(4‐nitrophenyl)methoxy]phenyl]methyl]‐4‐thiazolidinecarboxylic acid ethyl ester
YM‐244769: *N*‐[(3‐aminophenyl)methyl]‐6‐[4‐[(3‐fluorophenyl)methoxy]phenoxy]‐3‐pyridinecarboxamide dihydrochloride

**What is already known**

- A host of NCX blockers has been developed and studied in tissues other than glioblastoma.

**What this study adds**

- Blocking the forward but not reverse NCX can suppress glioblastoma growth via Ca^2+^‐mediated injury.

**What is the clinical significance**

- The NCX is a potential therapeutic target, and bepridil could be a leading compound.
- Summarized in 50 words, the significance of the work in language can be understood by a first‐year university science student, including what this paper adds to existing literature.
- Existing literature shows that the Na^+^/Ca^2+^ exchanger (NCX) and its blockers have been studied in tissues other than glioblastoma. We here found that blocking the forward NCX with bepridil can kill glioblastoma. We suggest that the NCX is a potential therapeutic target and bepridil could be a lead compound for this strategy.

 

## 1 INTRODUCTION

Intracellular http://www.guidetopharmacology.org/GRAC/LigandDisplayForward?tab=summary&ligandId=707 signalling plays an important role in cell fate determination such as quiescence or proliferation, survival, or death and, therefore, is receiving increasing attention in the study of glioblastoma evolution (Leclerc et al., 2016; Morrone, Gehring, & Nicoletti, 2016). Tumour biology studies document that glioblastoma cells release toxic concentrations of glutamate to surrounding neurons (Buckingham et al., 2011; Ye & Sontheimer, 1999). Glutamate constantly activates glutamate receptors in glioblastoma cells and causes Ca^2+^ influx. If Ca^2+^ extrusion mechanisms are inhibited, the intracellular Ca^2+^ ([Ca^2+^]_i_) will build up in glioblastoma cells. The accumulation of [Ca^2+^]_i_ is a well‐known step that causes ischaemic/hypoxic cell death (Dong, Saikumar, Weinberg, & Venkatachalam, 2006; Trump & Berezesky, 1995). An elevation of [Ca^2+^]_i_ may also be lethal to tumour cells and become a new approach for combating glioblastoma. However, the data regarding how to induce [Ca^2+^]_i_ overload and Ca^2+^‐dependent injury in glioblastoma are very limited (Hsu et al., 2016; Liang & Lu, 2012).

The http://www.guidetopharmacology.org/GRAC/FamilyDisplayForward?familyId=180 is an anti‐porter located in the plasma membrane. NCX participates in maintaining intracellular Ca^2+^ homeostasis by working either in the forward mode (Ca^2+^ extrusion) or in the reverse mode (Ca^2+^ entry) depending on the membrane potential and the transmembrane Ca^2+^ and Na^+^ concentrations (Giladi, Tal, & Khananshvili, 2016; Nagy et al., 2004). The mammalian NCX (SLC8) family contains three isoforms, NCX1, NCX2, and NCX3. NCX1 predominately exists in neurons, glial cells, cardiomyocytes, and renal tissue, while NCX2–NCX3 barely appear in tissues other than brain and skeletal muscle (Annunziato, Pignataro, & Di Renzo, 2004). NCX and the plasma membrane http://www.guidetopharmacology.org/GRAC/FamilyDisplayForward?familyId=159 are the main Ca^2+^ transporters that are in charge of extruding [Ca^2+^]_i_ in neurons and glial cells (Blaustein, Juhaszova, Golovina, Church, & Stanley, 2002). The NCX has 20‐fold to 60‐fold higher Ca^2+^‐transporting capacity than plasma membrane Ca^2+^–ATPase (Herchuelz, Kamagate, Ximenes, & Van Eylen, 2007), so NCX is the primary means of Ca^2+^ extrusion when [Ca^2+^]_i_ accumulates above the basal level. Inhibition of the NCX occludes the main route of Ca^2+^ extrusion and thus will result in [Ca^2+^]_i_ overload. To validate this speculation, we examined whether NCX blockers can elevate [Ca^2+^]_i_ in glioblastoma cells and induce Ca^2+^‐dependent cell death.

Over the past 20 years, many NCX blockers have been developed and employed to study the pathological role of NCX in hypoxic/ischaemic brain, heart, and renal tissue (Pignataro, Gala, et al., 2004; Shenoda, 2015). http://www.guidetopharmacology.org/GRAC/LigandDisplayForward?ligandId=2337 is often used as a forward NCX blocker in pharmacological studies; however, it also partially inhibits the reverse NCX and affects some K^+^ and Ca^2+^ channels in animal cardiomyocytes (Annunziato et al., 2004; Ma, Takanari, Masuda, Morishima, & Ono, 2016; Wang, Kiyosue, Kiriyama, & Arita, 1999; Watanabe & Kimura, 2001). http://www.guidetopharmacology.org/GRAC/LigandDisplayForward?tab=biology&ligandId=4593 is a more specific blocker for inhibiting the forward mode of NCX (Amoroso et al., 1997; Annunziato et al., 2004). http://www.guidetopharmacology.org/GRAC/LigandDisplayForward?ligandId=4232 is a blocker that inhibits the NCX in both operation modes (Watano, Harada, Harada, & Nishimura, 1999). Inhibition of forward NCX by bepridil aggravates ischaemic brain damage, whereas inhibitors that selectively target the reverse NCX, such as http://www.guidetopharmacology.org/GRAC/LigandDisplayForward?ligandId=4617, http://www.guidetopharmacology.org/GRAC/LigandDisplayForward?tab=summary&ligandId=4666, and http://www.guidetopharmacology.org/GRAC/LigandDisplayForward?ligandId=9484, produce protection in various hypoxic/ischaemic cell injury models (Hu & Song, 2017; Pignataro, Tortiglione, et al., 2004). Thus, pharmacological effects of NCX blockers rely on what mode of NCX operation is blocked at a given time. Theoretically, inhibition of the reverse NCX impedes NCX‐mediated Ca^2+^ entry and reduces Ca^2+^‐mediated injury. In contrast, blockade of the forward NCX (Ca^2+^ efflux) will lead to Ca^2+^ accumulation and cytotoxicity. Given that NCX blockers have been well developed and studied, it is timely and of great interest to know what action these NCX blockers will exert on glioblastoma. Particularly, involvement of the NCX in glioblastoma growth has not been well studied using either pharmacological or genetic approaches (Amoroso et al., 1997; Hsu, Chou, & Chueh, 1995).

Here, we showed that blocking reverse NCX with its selective inhibitors does not affect tumour growth; the glioblastoma is only suppressed when the forward NCX is blocked. Blocking the forward NCX is an effective strategy to beat glioblastoma. Therefore, the plasma membrane NCX may represent a new potential therapeutic target.

## 2 METHODS

### 2.1 Animals and ethical statement

Male athymic BALB/c nude mice were provided by Shanghai Laboratory Animal Center (Chinese Academy of Sciences, Shanghai, China). BALB/c nude mouse was developed through crosses and backcrosses between BALB/cABom‐nu and BALB/cAnNCrj‐nu at Charles River Laboratories Japan, Inc.; this mouse is inbred, and genetic monitoring results confirm it to be a BALB/c nude. The animal lacks a thymus, is unable to produce T cells, and is therefore immunodeficient. Mice were housed in the specific pathogen‐free animal facility with free access to food and water and a 12‐hr light to dark cycle. Mice at 8 weeks of age were chosen for experiments. Animal studies are reported in compliance with the ARRIVE guidelines (Kilkenny, Browne, Cuthill, Emerson, & Altman, 2010) and with the recommendations made by the *British Journal of Pharmacology*. All animal experimental procedures were approved by the Animal Experimentation Ethics Committee and Institutional Animal Care and Use Committee at Shanghai Jiao Tong University School of Medicine and carried out strictly in accordance with the guideline of Association for Assessment and Accreditation of Laboratory Animal Care. Efforts were made to minimize animals' suffering and to reduce the number of animals used.

### 2.2 Culture of glioblastoma cells and astrocytes

Human glioblastoma cell lines U87 (Cat# HTB‐14, RRID:CVCL_0022), A172 (Cat# CRL‐1620, RRID:CVCL_0131), and U118 (Cat# HTB‐15, RRID:CVCL_0633) were from American Type Culture Collection (Rockville, MD, USA). The glioblastoma cell line U251 (Cat# TCHu 58; Zhang et al., 2013) was from the Cell Bank of Type Culture Collection of the Chinese Academy of Sciences (Shanghai, China). The paediatric glioblastoma cell SF188 (Bender et al., 2013) was kindly provided by Dr Yu‐Jie Tang (Shanghai Jiao Tong University School of Medicine, China) and Dr Stefan Pfister (DKFZ, Germany). These cells were cultured in DMEM (Gibco, Carlsbad, CA, USA) containing l‐glutamine and 10% FBS (Gibco). U87 cells expressing the firefly luciferase gene (U87‐luciferase [U87‐Luc], Cat# CBP30189L, RRID:CVCL_UR33) were purchased from Nanjing COBIOER biotechnology company (Nanjing, China) and cultured in DMEM + 10% FBS + 1 μg·ml^−1^ puromycin. They were used at less than 30 passages. Human astrocytes (Cat# 1800) were purchased from ScienCell Research Laboratories (Carlsbad, CA, USA) and cultured in human astrocyte medium supplemented with 10% FBS and astrocyte growth supplement. After selecting for astrocytes in passage 1, passages 2–6 of human astrocytes were used for experiments. All cells were maintained at 37°C in an incubator with a humidified atmosphere containing 5% CO_2_ and 95% air.

### 2.3 Cell viability assay

Cell viability was determined by using a Cell Counting Kit‐8 (Dojindo, Japan). Briefly, cells were harvested from flasks and plated in 96‐well cell culture plates at 5 × 10^3^ per well and cultured overnight. Cells were treated with test reagents for 72 hr. Control cells were treated with cell culture medium and measured at the same time point as treatment groups. CCK‐8 solution (10 μl) was added to each well of the 96‐well plates, and the cultures were incubated for 30 min at 37°C. Absorbance at 450 nm was measured using an automatic microplate reader (Molecular Devices, San Jose, CA, USA).

### 2.4 Whole‐cell patch‐clamp recording

Whole‐cell patch‐clamp recording was performed using a Multiclamp 700B amplifier (Molecular Devices, Foster City, CA, USA) at room temperature. Voltage protocols were provided and current recorded via a DigiData‐1550B interface (Molecular Devices) controlled by pClamp 10 Software (Molecular Devices, RRID:SCR_011323). The external solution had a pH of 7.4 and contained (mM): 135 NaCl, 1.25 NaH_2_PO_4_, 1 MgCl_2_, 2 CaCl_2_, 10 HEPES, 10 glucose, 0.001 http://www.guidetopharmacology.org/GRAC/LigandDisplayForward?ligandId=2616, 0.02 http://www.guidetopharmacology.org/GRAC/LigandDisplayForward?ligandId=4826, and 0.01 http://www.guidetopharmacology.org/GRAC/LigandDisplayForward?ligandId=2514. Internal solution consisted of (in mM): 120 CsCl, 20 NaCl, 2 MgCl_2_, 13 CaCl_2_, 2 Na_2_ATP, 20 BAPTA, and 10 HEPES at a pH 7.2. Recording electrodes pulled from borosilicate glass pipettes (Sutter Instrument, Novato, CA, USA) had a tip resistance between 6 and 8 MΩ when filled with the internal solution. Series resistance was compensated by 75–85%. Linear leak and residual capacitance currents were subtracted online using a P/4 protocol of the pClamp 10. For the recording of the membrane currents related to the NCX, cells were held at −50 mV and depolarized to +60 mV, and then a voltage ramp starting from +60 to −120 mV was applied for 1,000 ms before returning back to −50 mV. The reverse and forward NCX currents were measured at +50 and −110 mV holding potentials respectively (Molinaro et al., 2008; Rahman, Inman, Kiss, & Janssen, 2012; Song, Chen, & Yu, 2014). Data were filtered at 3 KHz and digitized at sampling rates of 20 KHz. For drug application, cells were perfused with a VC‐6 Six Channel Perfusion Valve Control Systems (Warner Instruments, Greenwich, CT, USA) connected to a gravity‐fed perfusion systems.

### 2.5 Ca^2+^ and Na^+^ imaging

Cells were cultured in 96‐well plates at 8 × 10^3^ per well and loaded with the cell membrane‐permeable Ca^2+^ dye Fluo‐4 AM (5 μM) in 100‐μl HEPES‐buffered solution for 50 min. The solution contained (mM): 135 NaCl, 3 KCl, 2 CaCl_2_, 1 MgCl_2_, 10 glucose, and 10 HEPES, supplemented with 1% FBS at pH 7.4. To prepare the Ca^2+^‐free solution, 2‐mM CaCl_2_ was replaced with 1‐mM EGTA in the HEPES‐buffered solution. The 96‐well plate containing the Fluo‐4 AM‐loaded cells was mounted in a Leica TCS SP8 confocal system (Leica Microsystems, Wetzlar, Germany). The confocal microscope armed with 488‐nm laser light was employed to excite the dynamic fluorescent signals in time and synchronously, while test regents were added to cells. To do Na^+^ imaging, Fluo‐4 AM was replaced with the cell membrane‐permeable Na^+^ dye Asante NaTrium Green‐2 AM (ANG‐2 AM) at 5 μM, which was excited by 514‐nm laser light. Visual inspection and fluorescent imaging were carried out at room temperature. The imaging data were analysed with the software https://leica-las-af-lite.software.informer.com/ 2.5 (Leica). The imaging traces shown are representative of at least three separate experiments; 12–18 cells were imaged in each experiment.

### 2.6 Cell cycle and apoptosis assay

U87 cells were seeded in six‐well plates (Corning, Corning, NY, USA) with 1 × 10^6^ cells per well and treated with NCX blockers, BAPTA‐AM, or MAPK inhibitors. Drug treatment was performed by the person who is blinded to the experimental groups. After treatment, cells were harvested in 15‐ml tubes and washed with cold sterile PBS. After being centrifuged, cells were fixed with 70% ethanol for 30 min at 4°C for at least 1 hr. For propidium iodide (PI) staining, cells were washed one time with PBS and then were treated with ribonuclease A (RNase; 100 μg·ml^−1^) for 30 min. PI (10 μg·ml^−1^) was added, and stained cells were kept in the dark at 4°C until analysis. The number of cells analysed for each sample was 10,000 events. FACS analysis was performed using the Coulter CytoFlexS flow cytometer (Beckman Coulter, CA, USA), and the percentage of cells in the G_0_/G_1_, S, and G_2_/M phases was determined using cell cycle analysis software, ModFit LT 5.0 (Verity Software House, Topsham, ME, USA). For the apoptosis assay, cells were harvested and washed twice with cold PBS and resuspended at a density of 1 × 10^6^ cells·ml^−1^ in 100 μl of binding buffer containing 5 μl of Annexin V–FITC and 5 μl of PI working solution (100 μg·ml^−1^). After incubation at room temperature for 15 min in the dark, 400 μl of binding buffer was added to each sample. Apoptosis was analysed by Coulter CytoFlexS flow cytometer (Beckman Coulter) for at least 10,000 events, and data were analysed with the software, CyExpert 2.0 (Beckman Coulter).

### 2.7 Western blot analysis

After treatment with bepridil for 0, 30, 60, and 90 min, cells were collected and lysed in RIPA buffer (Beyotime, Nanjing, China) supplemented with protease inhibitor (Beyotime). The total protein concentration of each sample was determined using the BCA Protein Assay Kit (Sangon Biotech, Shanghai, China). Equal protein extracts (45‐μg protein per lane) were separated by SDS‐PAGE and electrophoretically transferred to PVDF membranes (Millipore, Bedford, MA, USA). Then, the membranes were incubated with anti‐phosphorylated and total http://www.guidetopharmacology.org/GRAC/FamilyDisplayForward?familyId=514, http://www.guidetopharmacology.org/GRAC/FamilyDisplayForward?familyId=518, and http://www.guidetopharmacology.org/GRAC/FamilyDisplayForward?familyId=519 antibodies (1:1,000; Cell Signaling Technology) at 4°C overnight, followed by incubation with IRDye 680LT fluorescent secondary antibody (LI‐COR Biosciences, Lincoln, NE, USA). Proteins were visualized using the Odyssey Fc Imaging System (LI‐COR Biosciences, Lincoln, NE, USA). Mouse α‐tubulin antibody was used as protein loading controls. The immuno‐related procedures used comply with the recommendations made by the *British Journal of Pharmacology* (Alexander et al., 2018).

### 2.8 Nucleoprotein extraction

Adherent cells were washed twice with 10 ml Dulbecco's PBS. Then 1‐mM PMSF, 1‐mM DTT, and 10‐μl protease inhibitor cocktail (without EDTA) per 1 ml of hypotonic buffer, mix hypotonic buffer, were added and samples placed on ice for a few minutes. After which 0.6‐ml mixed hypotonic buffer was added per 10,000,000 cells, on ice, then cells scraped off, and samples transferred to 1.5‐ml EP tube, centrifuged for 5 min (4°C, 835× *g*), and the supernatant removed. The cell lysates were resuspended by adding 0.6‐ml hypotonic buffer, centrifuged for 5 min (4°C, 835× *g*), and the supernatant removed. The pellet was resuspended in 0.3 ml of lysis buffer (lysis buffer contained 1‐mM PMSF, 1‐mM DTT, and 10‐μl protease inhibitor cocktail per 1 ml of lysis buffer), incubated on ice for 20 min, and centrifuged for 10 min (4°C, 13,362× *g*). The supernatant was nuclear protein extraction.

### 2.9 U87‐luciferase xenograft model and treatment

The athymic BALB/c nude mice were anaesthetized by subjecting them to 3% isoflurane (RWD Life Science, Shenzhen, China) in a mixture of 30% O_2_ and 70% N_2_O. After induction of anaesthesia, 1.5% isoflurane was maintained, and body temperature was kept at 37 ± 0.5°C by a heating pad. The mouse head was maintained within a stereotactic frame (RWD Life Science), allowing a precise and reproducible injection. The coordinate of the injection site was 0.5 mm anterior and 2.5 mm right from the bregma and at a depth of 3 mm. U87‐Luc cells (COBIOER) were assessed by the trypan blue exclusion assay. Only cell suspensions with greater than 95% viability were used and implanted into the right striatum of nude mice. U87‐Luc cells (5 × 10^5^ in 5‐μl PBS) were inoculated into the right striatum of each mouse at a controlled flow rate of 0.5 μl·min^−1^, and the total injection volume was about 5 μl. Sixteen days after implantation, the mice were randomly divided into two groups: treated with vehicle (*n*  =  5) and treated with bepridil (*n*  =  5). Bepridil was first dissolved in ethanol (final volume 5%) and then added to a mixture of polyethylene glycol (50%) and 0.9% NaCl (45%). Mice received bepridil (50 mg·kg^−1^ body weight) or vehicle treatment once a day via oral gavage for 14 days. Drug treatment was performed by a person who was blinded to the experimental groups.

### 2.10 MS analysis

Athymic BALB/c nude mice were administered bepridil by oral gavage at doses of 50 mg·kg^−1^ as described above. Two hours later, mice were killed, and the brain sample was homogenized using the tissue homogenizers (Bertin Instruments, Paris, France) in ice‐cold deionized water at a concentration of 3 ml·g^−1^ tissue. Then, methanol with 0.1% formic acid was added to the brain homogenate in a 4:1 proportion (vol/vol) and centrifuged at 18,188× *g* and 4°C for 10 min to precipitate protein. The supernatant was transferred to a vial to be injected directly into the LC‐MS. The chromatographic system consisted of Shimadzu quaternary pump, vacuum degasser, and autosampler (Shimadzu, LC‐20ADXR, Tokyo, Japan) coupled to https://sciex.com/products/mass-spectrometers/triple-quad-systems, API‐4000 mass spectrometer (SCIEX, Redwood City, CA, USA). HPLC separation was performed on a Thermo Syncronis C8 column 100 × 2.1 mm, 5.0 μm. The mobile phase consisted of methanol (A) and water with 0.1% formic acid (B), measure time was 9 min per run. A flow rate of 0.3 ml·min^−1^ was used using a gradient elution of 70% B at 1 min and 5–70% B between 1 and 5 min and maintained for 2 min at 5% B and back to 70% B at 7–7.1 min. An API‐4000 mass spectrometer equipped with ion spray source was employed for obtaining mass spectra. Data acquisition was carried out by analysis software. Ion spray voltage was set at 5,500 V. Curtain gas was kept at 35 psi. Ion source temperature was 550°C. Nebulizing gas and drying gas were at 50 psi. Multiple reaction monitoring mode was utilized to detect the compound of interest. Collision energy (CE) is an instrument parameter that is frequently optimized to increase fragment ion intensity. An alternative to empirically optimizing the CE for bepridil is to predict the best CE value based on the precursor mass‐to‐charge ratio of bepridil (*m*/*z*). The CE for bepridil was optimized to 30 V. The precursor‐to‐product ions (Q1 → Q3) selected for bepridil during quantitative optimization were (*m*/*z*) 367.1 → 84.3/184.3.

### 2.11 In vivo bioluminescent imaging

Tumours were measured by bioluminescence using an IVIS Spectrum CT Imaging System (PerkinElmer, Waltham, MA, USA), which captured the luminescence signal emitted from the engrafted tumour. Prior to imaging, the mice were anaesthetized with inhalation of isoflurane gas (RWD Life Science) and injected i.p. with 150 mg·kg^−1^ d‐luciferin potassium (J&K Scientific) aqueous solution. The isoflurane was balanced with oxygen and dialled to 2.0% for the induction of anaesthesia and 1.0% for maintenance. Images were subsequently captured 10 min following injection. Signal intensity was quantified within a region of interested over the head, as defined by Living Image software. All images represent 2‐min exposure time, and the average number of photons·s^−1^·cm^−2^ per steradian were recorded. The data were analysed using Living Image 4.4.5 software (PerkinElmer, RRID:SCR_014247).

### 2.12 Data and statistical analysis

Data are expressed as mean ± SEM and analysed by Prism 7 software, La Jolla, CA (GraphPad Prism, RRID:SCR_002798). The data and statistical analysis comply with the recommendations of the *British Journal of Pharmacology* on experimental design and analysis in pharmacology. Concentrations of the NCX blockers (IC_50_) exerting half‐maximal inhibition of NCX currents were obtained by fitting the concentration–response with the equation: $I/I_{0}\;*\;100\%\;=\;\text{Bottom}\;\;+\left(\mathrm{Top}\;-\;\;\text{Bottom}\right)/\left(1+{{10^{((}}^{\mathrm{Log}}}^{{\mathrm{IC}}_{50}\;-\;C)\;\;*\;n)}\right)$, where *I*_0_ and *I* are current amplitudes measured in control and in the presence of NCX blockers, C is the logarithm of concentration, and *n* is the Hill coefficient. The statistical difference between two independent groups was analysed by Student's parametric unpaired *t* test. And the original data of more than two groups were assessed by the parametric one‐way ANOVA followed by a Tukey's post hoc test. The data for cell viability and Western blot were usually normalized to the control group, and were thus analysed by the non‐parametric Kruskal–Wallis test followed by a Dunn's post hoc test. Differences were considered to be significant when *P* < .05. Post hoc tests were conducted when the *F* value achieved the necessary level (*P* < .05) and there was no significant variance inhomogeneity.

### 2.13 Materials

[1,2‐Bis(2‐aminophenoxy)ethane‐*N*,*N*,*N*′,*N*′‐tetraacetic acid (BAPTA‐AM)] (Cat# 2787), YM‐244769 (Cat# 4544), KB‐R7943 (Cat# 1244), SN‐6 (Cat# 2184), SEA0400 (Cat# 6164), and bepridil (Cat# 4117) were purchased from Tocris Bioscience (Minneapolis, MN, USA). They were dissolved in DMSO to make a stock solution. Cell Counting Kit‐8 and Annexin V–FITC/PI Apoptosis Detection Kit were purchased from Dojindo Laboratories (Tokyo, Japan). Pyridinium iodide was purchased from Sigma‐Aldrich (St. Louis, MO, USA). Cell‐permeable Fluo‐4 AM was purchased from Invitrogen Life Technologies (Waltham, MA, USA). The primary antibodies for phosphorylated and total ERK (Cat# 8544 and 4348), JNK (Cat# 9251 and 9252), and p38‐MAPK (Cat# 9211 and 9212) were all purchased from Cell Signaling Technology (Boston, MA, USA). Anti‐NCX1 antibody (Cat# ab177952, RRID:AB_2801276) was purchased from Abcam (Cambridge, MA, USA), and anti‐NCX2 (Cat# ANX‐012, RRID:AB_2341022) and NCX3 (Cat# ANX‐013, RRID:AB_2341023) antibodies were purchased from Alomone Labs (Jerusalem, Israel). http://www.jkchemical.com/CH/products/A01433521.html J&K Scientific (Sunnyvale, CA, USA). JNK inhibitor (CC‐930, Cat# HY‐15495) and p38‐MAPK inhibitor (PD169316, Cat# HY‐10578) were purchased from http://www.baidu.com/baidu.php?url=060000jZYuibWkd0M6aReminygCYDcwtTwLjtyVWGcEcZs3lGVEKX9cQrKeEPTfy1RZ2Ey30HzY85wEMjq7fCSy7NUmoQvHU-rNyfSw4-baUKbGm_nngTM-v_msSXc6WniKv-yohiMG9naSfgZEt_CxX_TiVgzgEwqDM3X6us6mYtGmNN6.7D_jihlAGr-5wWhHZZzI34CyQe_qJknpgwRgkF4X5QIJyAp7BEGtxAVf.U1Yz0ZDqHRP7Y2ALdVxHYVUyko1K0ZKGm1Ys0ZfqUyP-FHcsYVUyko1K0A-V5HczPfKM5gK1IZc0Iybqmh7GuZN_UfKspyfqn6KWpyfqn1bv0AdY5HDsnHIxnH0krNtznjmzg1DsPW7xn1msnfKopHYs0ZFY5HbsP0K-pyfqnHfYnNtznH03P6KBpHYznjwxnHRd0AdW5HT4nH03rjckndtknj0kg1n1nWD4nj6sPj7xnH0zg100TgKGujYs0Z7Wpyfqn0KzuLw9u1Ys0A7B5HKxn0K-ThTqn0KsTjYLrjT3P1DzPHR0UMus5H08nj0snj0snj00Ugws5H00uAwETjYs0ZFJ5H00uANv5gKW0AuY5H00TA6qn0KET1Ys0AFL5HT0UMfqn0K1XWY0IZN15HDsPj0YPjfvn1TkPWRLn1D1PHmv0ZF-TgfqnHRYnj64nHcYrjnsP6K1pyfqmvD4uAc3ryRsnj0sryN9r0KWTvYqPWn1nRwDwWRsPYwDnYfdnfK9m1Yk0ZK85H00TydY5H00Tyd15H00XMfqn0KVmdqhThqV5HKxn7tsg1Kxn0Kbmy4dmhNxTAk9Uh-bT1Ysg1Kxn7tznW61nHfsg100TA7Ygvu_myTqn0Kbmv-b5H00ugwGujYVnfK9TLKWm1Ys0ZNspy4Wm1Ys0Z7VuWYkP6KhmLNY5H00uMGC5H00uh7Y5H00XMK_Ignqn0K9uAu_myTqnfK_uhnqn0KWThnqPW0LP6&ck=2697.17.1540891321553.0.0.134.151.0&shh=www.baidu.com&sht=monline_3_dg (Monmouth Junction, NJ, USA). Mouse α‐tubulin antibody (Cat# 2144) was purchased from Cell Signaling Technology. Ribonuclease A from bovine pancreas was purchased from Sigma‐Aldrich. Isoflurane gas was purchased from RWD Life Science (Shenzhen, China).

### 2.14 Nomenclature of targets and ligands

Key protein targets and ligands in this article are hyperlinked to corresponding entries in http://www.guidetopharmacology.org, the common portal for data from the IUPHAR/BPS Guide to PHARMACOLOGY (Harding et al., 2018), and are permanently archived in the Concise Guide to PHARMACOLOGY 2017/18 (Alexander, Fabbro et al., 2017; Alexander, Kelly et al., 2017).

## 3 RESULTS

### 3.1 Inhibition of the Na^+^/Ca^2+^ exchanger by its blockers in glioblastoma cells

**Figure 1:**
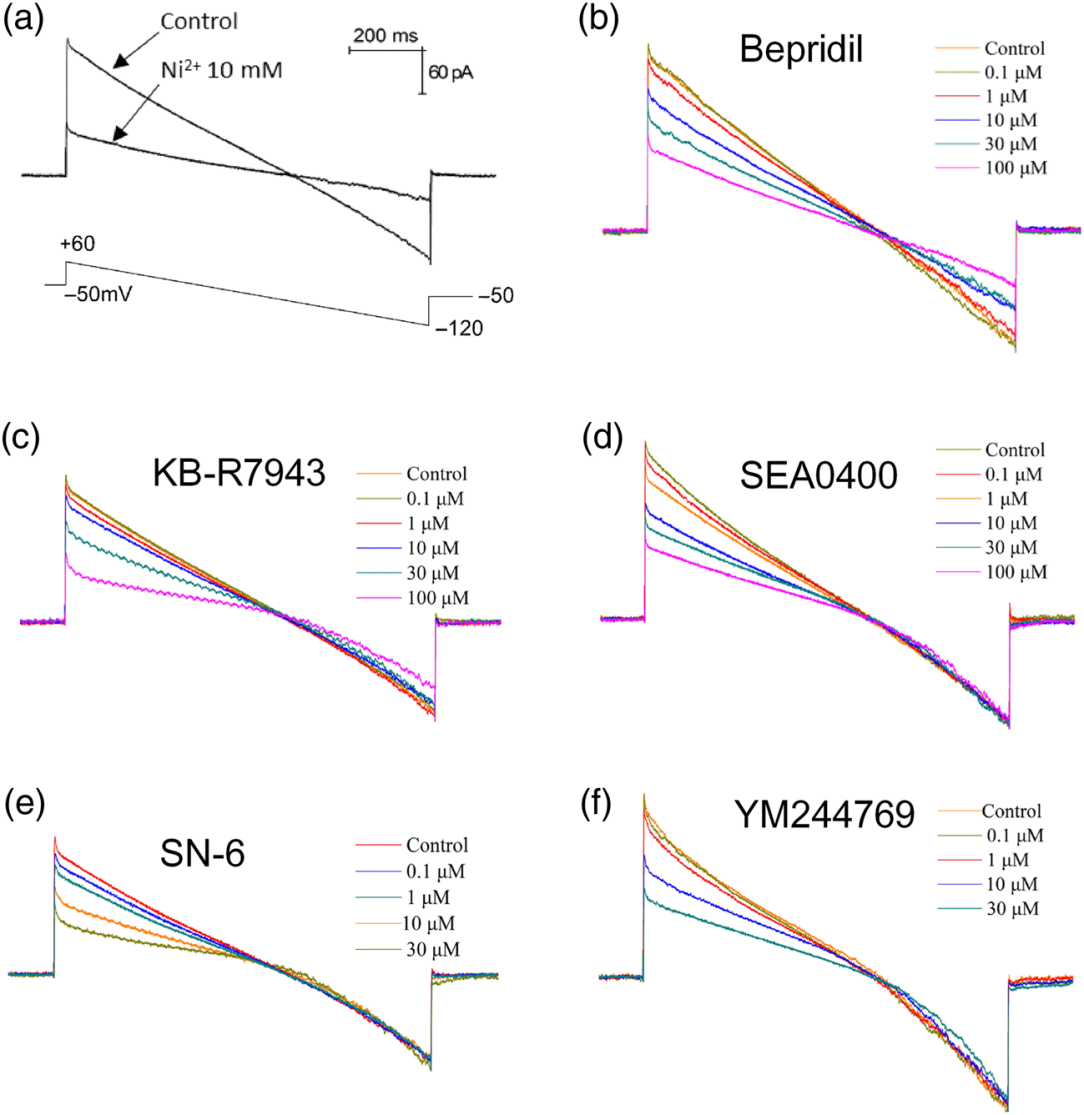
Recording and blockade of the Na^+^/Ca^2+^ exchanger (NCX) currents in glioblastoma cells. (a) Human glioblastoma U87 cells were held at −50 mV; the membrane currents associated with NCX were recorded by a voltage ramp from +60 to −120 mV. Ni^2+^ (10 mM) was used to block the NCX currents. (b, c) NCX currents recorded before and after application of bepridil (0.1–100 μM) or KB‐R7943 (0.1–100 μM). (d–f) The currents recorded before and after application of the reverse NCX blockers SEA0400 (0.1–100 μM), SN‐6 (0.1–30 μM), and YM244769 (0.1–30 μM). The reverse NCX current was measured at +50 mV, and forward NCX current was measured at −110 mV

We characterized the membrane currents associated with NCX activity in glioblastoma cells using whole‐cell patch‐clamp recording. As illustrated in Figure 1a, cell membrane potential was held at −50 mV, and the NCX currents were elicited by a voltage ramp from +60 to −120 mV. The NCX current in its reverse mode (Ca^2+^ entry) was measured at +50, and the forward‐mode current (Ca^2+^ exit) was measured at −110 mV. The Ni^2+^ (10 mM) was employed to inhibit and identify the NCX currents in its either reverse or forward mode as previously reported (Maack, Ganesan, Sidor, & O'Rourke, 2005). The reversal potential of NCX current is around −60 mV determined by intersection of current–voltage relation (*I*/*V*) in the absence and presence of Ni^2+^. Bepridil has been reported to block forward‐mode NCX and also partially inhibits its reverse mode (Annunziato et al., 2004). We found bepridil blocked both direction of NCX currents in U87 cells, with half‐maximal inhibition (IC_50_) values of 19.1 ± 1.2 and 15.6 ± 1.1 μM (Figure 1b and Table 1). KB‐R7943 preferentially inhibits the reverse NCX, and it also blocks the forward NCX (Annunziato et al., 2004). We found KB‐R7943 inhibited the reverse NCX currents with an IC_50_ value of 14.3 ± 1.2 μM, while suppressed its forward currents with an IC_50_ = 32.3 ± 1.3 μM (Figure 1c and Table 1). SEA0400, SN‐6, and YM244769 have good selectivity for the reverse NCX (Figure 1d–f). They selectively block the reverse mode currents of NCX with IC_50_ values of 3.6 ± 1.2, 3.2 ± 1.2, and 4.5 ± 1.1 μM, without inhibition of the forward NCX currents.

**Table 1:** Concentration of compounds required to produce half‐maximal inhibition (IC_50_: μM) of the currents related to the reverse and forward NCX operation

Compound	Blocking reverse NCX	Blocking forward NCX	*n*
Bepridil	15.6 ± 1.1	19.1 ± 1.2	4
KB‐R7943	14.3 ± 1.2	32.3 ± 1.3	4
SEA0400	3.6 ± 1.2	—	4
SN‐6	3.2 ± 1.2	—	4
YM244769	4.5 ± 1.1	—	4

*Note*. The values of IC_50_ (mean ± SEM) were obtained by fitting the concentration–response with the equation: $I/I_{0}*100\%=\text{Bottom}+\left(\mathrm{Top}-\text{Bottom}\right)/\left(1+{{10^{((}}^{\mathrm{Log}}}^{{\mathrm{IC}}_{50}-C)*n)}\right)$, where *I*_0_ and *I* are current amplitudes measured in control and in the presence of NCX blockers, C is the logarithm of concentration, and *n* is the Hill coefficient. *n* = 4 independent tests for each compound.

Abbreviation: NCX, Na^+^/Ca^2+^ exchanger.

### 3.2 Blocking the forward NCX operation generates cytotoxicity in glioblastoma cells

**Figure 2:**
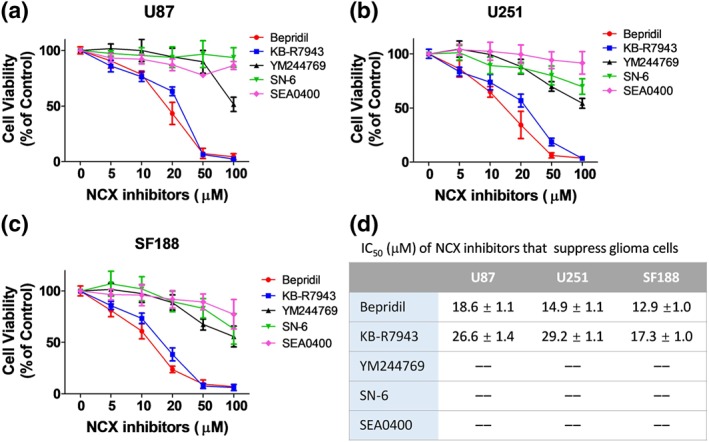
Effect of the Na^+^/Ca^2+^ exchanger (NCX) blockers on viability of human glioblastoma cell lines. (a, b) Viability of adult glioblastoma cell lines (U87 and U251) after exposure to NCX blockers bepridil, KB‐R7943, SEA0400, SN‐6, or YM244769 at 0–100 μM for 72 hr. *n* = 5 independent tests per group. (c) Viability of paediatric glioblastoma cell line SF188 after 72‐hr incubation with the same NCX blockers at 0–100 μM. *n* = 5 independent tests per group. (d) For U87, U251, and SF188 cell lines, concentration of the NCX blockers required to produce half maximal inhibition (IC_50_: μM, mean ± SEM) of cell viability. IC_50_ was obtained by fitting the normalized concentration–response relationship to the equation: *V*/*V*_0_   =   1/{1 +  [C/IC_50_]^*n*^}, where *V*_0_ and *V* are the cell viability measured in control and in the presence of a blocker, C is the concentration of the blocker, and *n* is the Hill coefficient. *n* = 5 independent cultures in each group

We next examined impact of NCX blockers on viability of glioblastoma cells. U87 and U251 cell lines were treated with bepridil or KB‐R7943 for 72 hr. We found that viability of U87 and U251 cells was reduced to 30–40%, 6–7%, and 3–4% of control level by 20‐, 50‐, and 100‐μM bepridil (Figure 2a,b). Similarly, U87 and U251 cells were regressed to 50–60%, 7–18%, and 2–3% of control level by 20‐, 50‐, and 100‐μM KB‐R7943. Figure 2d lists IC_50_ values of bepridil and KB‐R7943 that cause cytotoxicity. Since bepridil and KB‐R7943 inhibit both forward and reverse NCX, we need to clarify whether this cytotoxicity resulted from blocking the reverse or forward NCX operation.

To do so, three inhibitors (SEA0400, SN‐6, and YM244769) selective for the reverse NCX were incubated with U87 and U251 cells at 5, 10, 20, 50, and 100 μM for 72 hr. We found that they exert much less impact on glioblastoma cells (Figure 2a,b). SEA0400 at 10, 20, and 50 μM modulated U87 and U251 cell viability to 93–106%, 88–103%, and 72–92% of control level. SN‐6 at 10, 20, and 50 μM modulated U87 and U251 cells to of 87–97%, 86–99%, and 74–104% of control level. YM244769 at 10, 20, and 50 μM suppressed U87 and U251 cells to 87–100%, 84–96%, and 71–76% of control level. So, the growth of glioblastoma cells is not affected when the reverse NCX is blocked.

We also applied NCX blockers to a paediatric glioblastoma cell line SF188. These cells were suppressed by bepridil and KB‐R7943 in a dose‐dependent manner but slightly affected by the compounds that selectively block the reverse NCX (Figure 2c). CB‐DMB is a more specific blocker for the forward NCX than bepridil and KB‐R7943. It is very exciting to find that CB‐DMB is more potent than bepridil and KB‐R7943 in suppressing glioblastoma cells (Figure S1a). The IC_50_ values of CB‐DMB that cause cytotoxicity in U87, U251, and SF188 are 3.1 ± 0.6, 2.7 ± 0.1, and 2.6 ± 0.1 μM. These data confirm that blocking the forward but not reverse mode of NCX can suppress the growth of glioblastoma cells.

### 3.3 Bepridil, CB‐DMB, and KB‐R7943 elevate [Ca^2+^]_i_ level in glioblastoma cells

**Figure 3:**
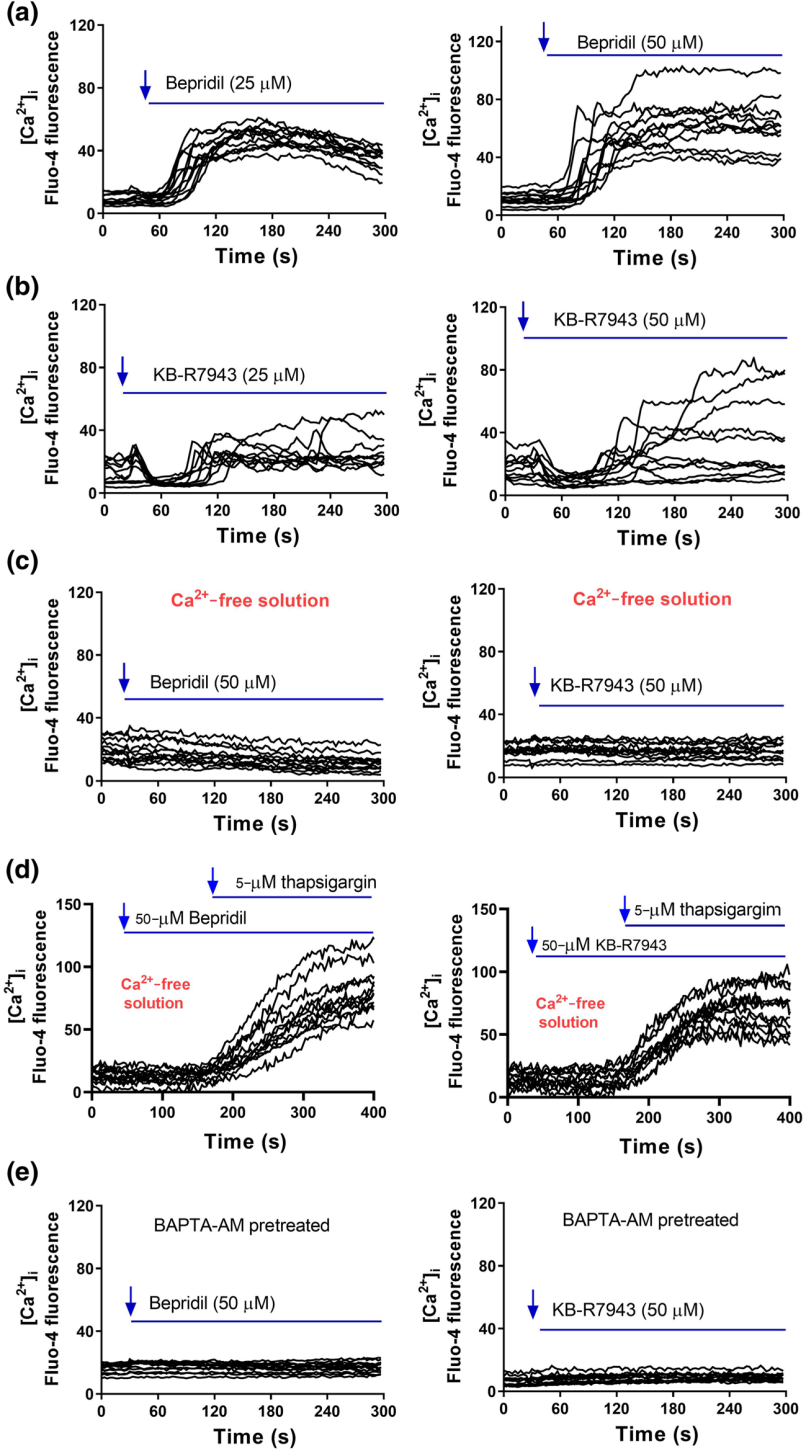
Effect of bepridil and KB‐R7943 on the level of [Ca^2+^]_i_ measured by Ca^2+^ imaging. (a) Flua‐4 AM‐loaded U87 cells were imaged and perfused with bepridil (25 or 50 μM) in the HEPES‐buffered solution containing 2‐mM Ca^2+^. (b) Ca^2+^ imaging of U87 cells before and after perfusion with KB‐R7943 (25 or 50 μM). (c) In the Ca^2+^‐free solution, Ca^2+^ imaging of U87 cells before and after perfusion with bepridil or KB‐R7943. (d) Ca^2+^ imaging before and after perfusion with bepridil or KB‐R7943, followed by application of 5‐μM thapsigargin. (e) U87 cells were pretreated with a Ca^2+^ chelator BAPTA‐AM (20 μM) for 2 hr and then imaged and perfused with bepridil or KB‐R7943. The traces are representative of three separate experiments, and *n* = 12–18 cells were imaged per experiment

We examined influence of NCX blockers on [Ca^2+^]_i_ level in glioblastoma cells. U87 cells were pre‐loaded with Fluo‐4 and then perfused with bepridil or KB‐R7943 in the solution containing 2‐mM Ca^2+^ and 1% FBS. Shortly after flush with bepridil, U87 cells showed a quick [Ca^2+^]_i_ increase and sustained [Ca^2+^]_i_ elevation (Figure 3a). KB‐R7943 induced two phases of [Ca^2+^]_i_ change: a short [Ca^2+^]_i_ descent in the beginning and a slow [Ca^2+^]_i_ increases ensuing (Figure 3b). To identify the source of bepridil and KB‐R7943‐induced [Ca^2+^]_i_ increase, we conducted the same Ca^2+^ imaging experiment in a Ca^2+^‐free solution. After perfusion with bepridil or KB‐R7943 (Figure 3c), glioblastoma cells did not show [Ca^2+^]_i_ elevation or fluctuation as that occurs in the solution containing Ca^2+^. Figure 3d shows that after bepridil or KB‐R7943, thapsigargin induced Ca^2+^ release from the endoplasmic reticulum (ER) in the Ca^2+^‐free solution. These data indicate that neither bepridil nor KB‐R7943 affect intracellular Ca^2+^ pool and Ca^2+^ transporting on the ER membrane.

Figure 3e shows that pretreated with Ca^2+^ chelator, BAPTA‐AM (20 μM) completely abrogated the influence of bepridil and KB‐R7943 on [Ca^2+^]_i_. Similarly, CB‐DMB elevated [Ca^2+^]_i_ in the Ca^2+^‐containing solution and did not affect the ER Ca^2+^ transporters (Figure S1b–d). The Na^+^ imaging experiment with Na^+^ indicator Asante NaTRIUM Green™ 2 (ANG‐2) shows that bepridil and CB‐DMB did not change the level of intracellular Na^+^ [Na^+^]_i_, while KB‐R7943 slightly decreased [Na^+^]_i_ (Figure S2a–c). Finally, there are no detectable NCX isoforms in the nuclei of U87 cells (Figure S2d). Bepridil, CB‐DMB, and KB‐R7943 all block the forward NCX on cell membrane; this blockade occludes NCX‐mediated Ca^2+^ exit. On the other hand, extracellular Ca^2+^ can still enter a cell through other Ca^2+^‐permeable channels, leading to accumulation of [Ca^2+^]_i_. The transient drop of [Ca^2+^]_i_ caused by KB‐R7943 may result from its blocking action on glutamate receptor channels as previously reported (Brustovetsky et al., 2011).

### 3.4 Bepridil, CB‐DMB, and KB‐R7943 cause cell cycle arrest and apoptosis

**Figure 4:**
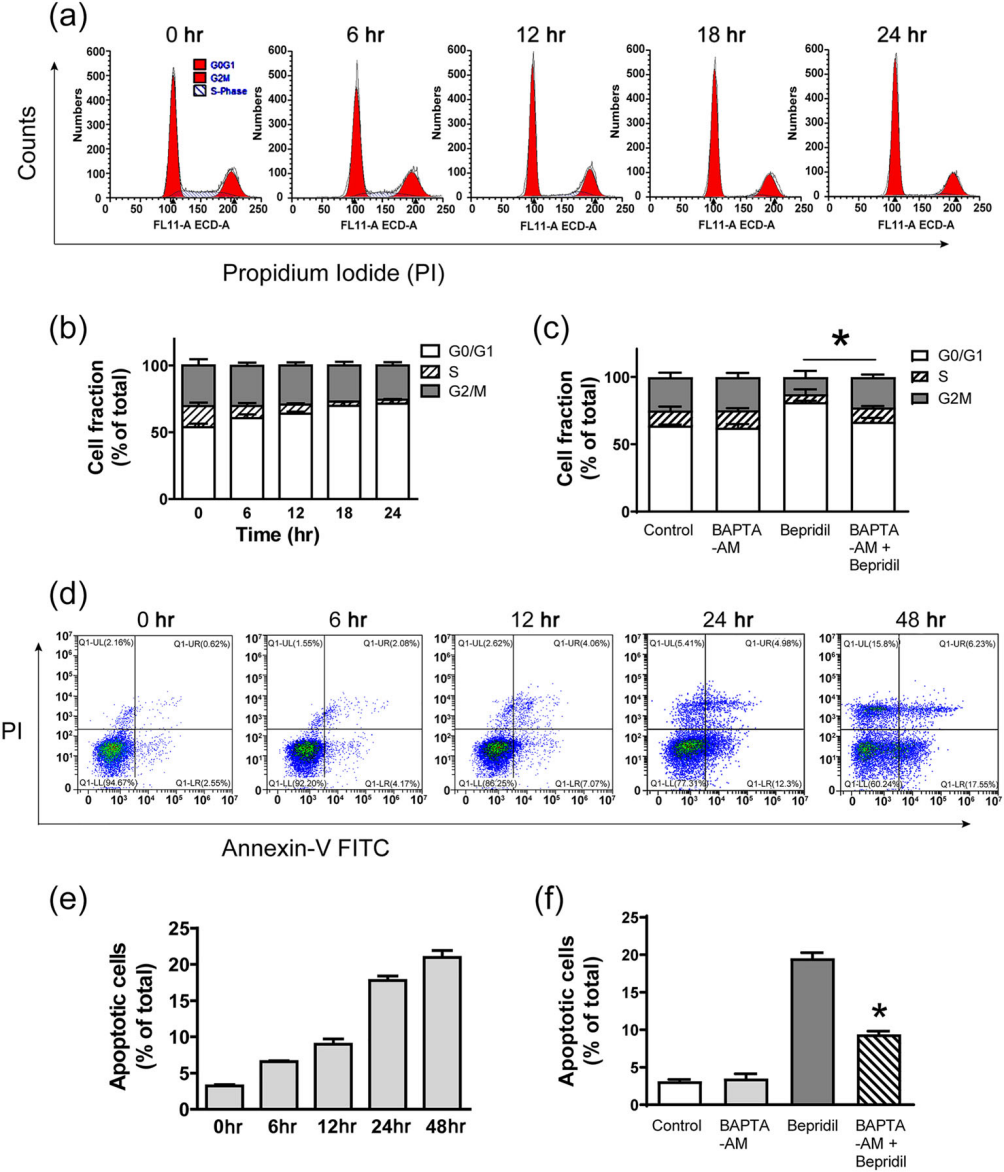
Bepridil caused cell cycle arrest and apoptosis in glioblastoma cells. (a, b) Cell cycle assay of U87 cells incubated with bepridil (25 μM) for 6, 12, 18, and 24 hr. Cell fractions in each phase were from cell cycle analysis; bepridil increased the proportion of cells in G_0_/G_1_ phase and reduced cell fraction in S phase in a time‐dependent manner. (c) Cells were pretreated with BAPTA‐AM (10 μM) for 18 hr and then incubated with bepridil (25 μM) for 18 hr. BAPTA‐AM alone did not incur cell fraction changes but significantly attenuated bepridil‐induced cell cycle arrest. *P < .05, G_0_/G_1_ phase of BAPTA‐AM + bepridil versus bepridil alone; *n* = 5 independent experiments. (d, e) U87 cells were treated with bepridil (25 μM) for 6, 12, 24, and 48 hr and then analysed with Annexin V–FITC/PI apoptosis assay. Bepridil incurred a time‐dependent increase of apoptotic cells (Annexin V^+^). (f) Cells were pretreated with BAPTA‐AM (10 μM) for 18 hr and then incubated with bepridil (25 μM) for 24 hr. BAPTA‐AM significantly suppressed bepridil‐induced apoptosis. *n* = 5 independent experiments; *P < .05, apoptotic cell fraction in BAPTA‐AM + bepridil versus bepridil alone, with the parametric one‐way ANOVA followed by Tukey's post hoc test

To do cell cycle analysis, U87 cell cultures were treated with 25‐μM bepridil for 6, 12, 18, and 24 hr and stained with PI. We show that bepridil reduced the cell fraction in S phase and increased cells in G_0_/G_1_ phase in a time‐dependent manner (Figure 4a,b). To determine the role of [Ca^2+^]_i_ in bepridil‐induced G_0_/G_1_ arrest, BAPTA‐AM was used to prevent [Ca^2+^]_i_ accumulation in U87 cells. Figure 4c shows that BAPTA‐AM (10 μM) alone for 18 hr did not incur significant cell fraction changes in G_0_/G_1_, S and G_2_/M phases. Bepridil (25 μM, 18 hr) markedly increased cell fraction in G_0_/G_1_ phase. This G_0_/G_1_ arresting effect was significantly attenuated by 10‐μM BAPTA‐AM (Figure 4c). To detect any apoptotic cell death involved, U87 cells were treated with bepridil (25 μM) for 6, 12, 24, and 48 hr and then analysed with Annexin V–FITC/PI apoptosis assay. Figure 4d,e shows that bepridil incurred a time‐dependent increase of apoptotic (Annexin V^+^) cells. BAPTA‐AM (10 μM) alone for 18 hr did not increase apoptosis in U87 cells but significantly attenuated bepridil‐induced apoptosis (Figure 4f). Application of CB‐DMB (3.5 μM) to U87 cells also induced G_0_/G_1_ arrest and activated apoptosis in time‐dependent manner (Figure S3a,b,d–e), and these cell death processes were significantly attenuated by BAPTA‐AM (Figure 3c,f). These data indicate that bepridil and CB‐DMB generate cytotoxicity through Ca^2+^‐mediated cell cycle arrest and apoptosis in glioblastoma cells.

**Figure 5:**
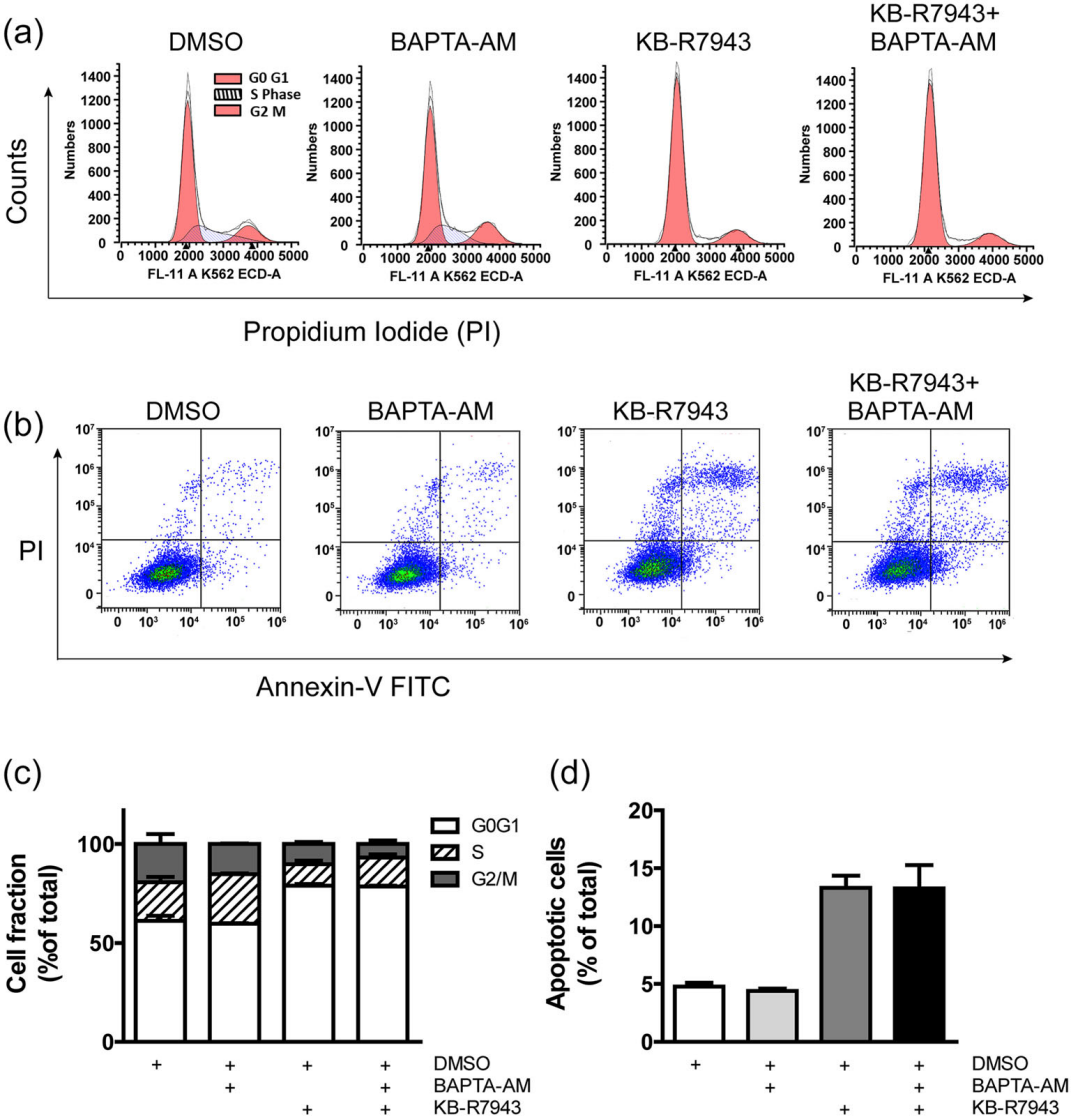
KB‐R7943 caused cell cycle arrest and apoptosis in glioblastoma cells. (a) U87 cells were incubated with 0.1% DMSO (vol/vol), BAPTA‐AM (10 μM), KB‐R7943 (25 μM), and BAPTA‐AM + KB‐R7943 for 18 hr and then underwent cell cycle analysis. (b) U87 cells underwent an apoptosis assay after incubation with 0.1% DMSO, 10‐μM BAPTA‐AM, 25‐μM KB‐R7943, and BAPTA‐AM + KB‐R7943 for 20 hr. (c) Cell fractions in G_0_/G_1_, S, and G_2_/M phases of each group. KB‐R7943‐induced G_0_/G_1_ arrest was not rescued by BAPTA‐AM. *n* = 5 independent experiments; data were analysed by one‐way ANOVA. (d) Percentage of apoptotic cells in each group. KB‐R7943‐induced apoptosis was not attenuated by BAPTA‐AM. *n* = 5 independent experiments; data were analysed by one‐way ANOVA

KB‐R7943 also caused G_0_/G_1_ arrest and apoptosis in U87 cells, but Ca^2+^ chelator BAPTA‐AM cannot suppress these cell death processes (Figure 5a–d). We notice that KB‐R7943 is not a pure NCX blocker (Cheng, Zhang, Du, Dempsey, & Hancox, 2012); it also inhibits ionotropic glutamate NMDA receptors and mitochondrial respiratory complex 1 (Brustovetsky et al., 2011). KB‐R7943‐induced cytotoxicity does not simply result from NCX inhibition and [Ca^2+^]_i_ elevation. Inhibition of respiratory chain complex 1 impairs mitochondrial oxidative phosphorylation and results in energy deficiency, which might contribute to the cytotoxicity of KB‐R7943. Pharmacological action of KB‐R7943 in this and past studies may need revisiting and re‐evaluation in future.

### 3.5 Involvement of MAPK signalling in bepridil‐induced cell death

**Figure 6:**
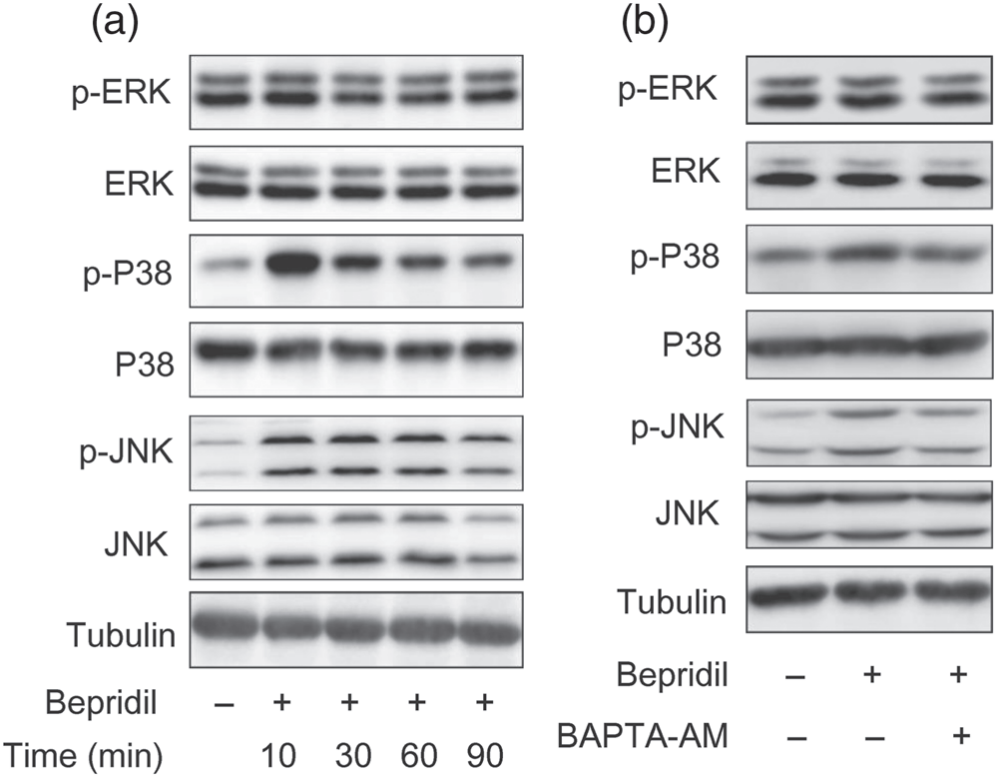
Bepridil induced activation of p38‐MAPK and JNK signalling. (a) U87 cells were treated with bepridil at 25 μM for 10, 30, 60, and 90 min. The phosphorylated and total ERK, p38‐MAPK, and JNK were detected by Western blot. Tubulin was an internal control. (b) Cells were pretreated with BAPTA‐AM (10 μM) for 18 hr and then incubated with bepridil (25 μM) for 30 min. Activity of the p38‐MAPK and JNK signalling was enhanced by bepridil but was attenuated in the presence of BAPTA‐AM

The above data indicate that bepridil killed glioblastoma cells via blockade of the forward NCX and elevation of [Ca^2+^]_i_. It is well known that [Ca^2+^]_i_ regulates the MAPK signalling pathway, which plays essential role in cell fate determination (White & Sacks, 2010). We here measured the activities of ERK, p38‐MAPK, and JNK in U87 cells at the time points 10, 30, 60, and 90 min after incubation with 25‐μM bepridil. Western blot analysis shows that ERK activity was not affected, but p38‐MAPK and JNK were activated by bepridil throughout the selected time points (Figure 6a). To examine the role of [Ca^2+^]_i_ in bepridil‐induced MAPK activation, cells were pretreated with Ca^2+^ chelator BAPTA‐AM (10 μM) for 18 hr and then exposure to bepridil (25 μM) for 30 min. Figure 6b shows that activity of the p38‐MAPK and JNK was increased by bepridil, and this effect was attenuated by BAPTA‐AM pretreatment.

**Figure 7:**
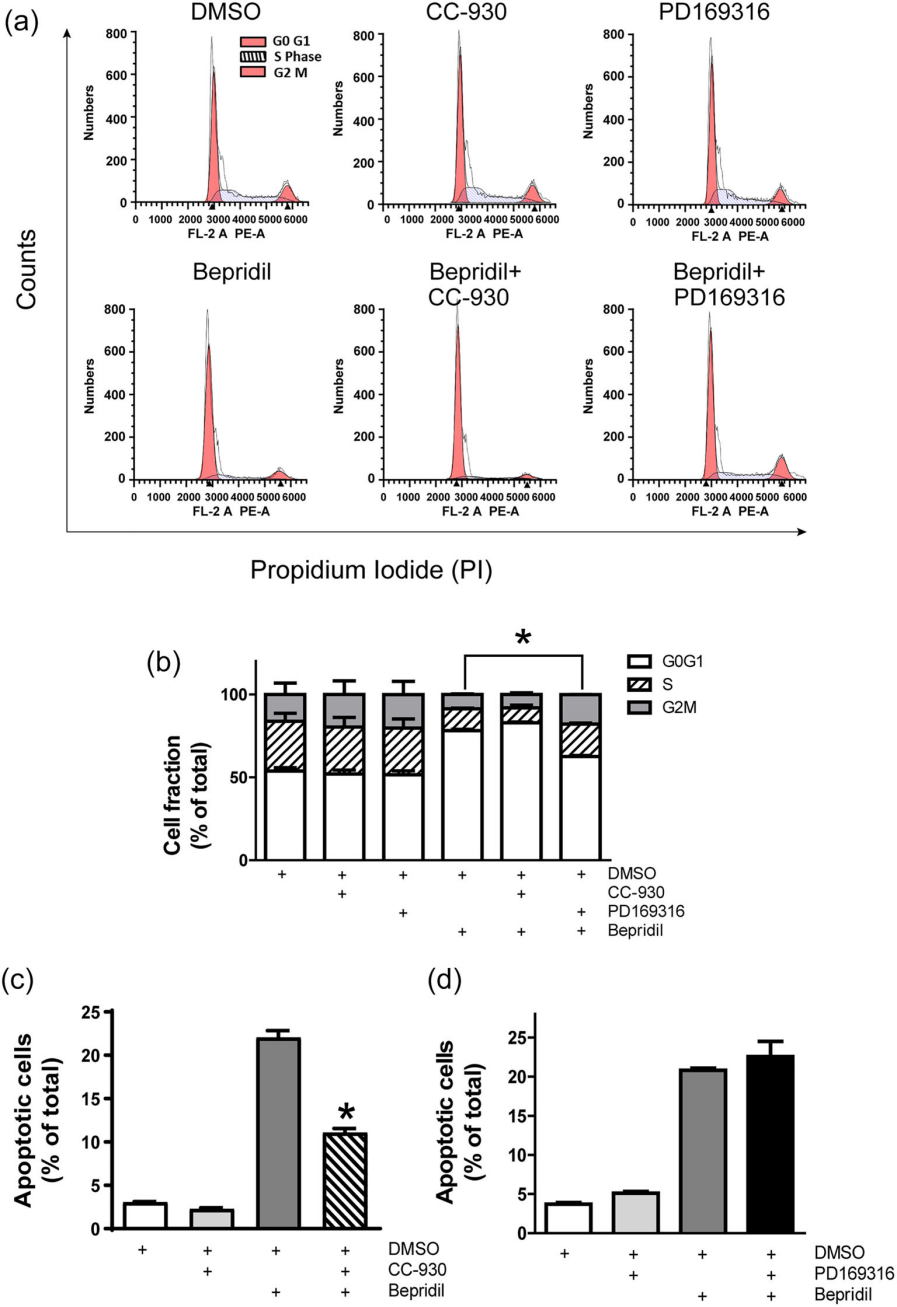
Involvement of MAPK signalling in bepridil‐induced cell cycle arrest and apoptosis. (a) Top panel, U87 cells were treated with DMSO (0.1/%, vol/vol), CC‐930 (10 μM), and PD169316 (10 μM) for 18 hr. Lower panel, cells were treated with bepridil (25 μM) alone or pretreated with CC‐930 (10 μM) and PD169316 (10 μM) for 2 hr and then incubated with bepridil for 18 hr. (b) Cell fractions in G_0_/G_1_, S, and G_2_/M phases of each group. Bepridil‐induced G_0_/G_1_ cell cycle arrest was rescued by PD169316 but not CC‐930. *P < .05, cell fraction in G_0_/G_1_ phase of PD169316 + bepridil‐treated group versus bepridil‐treated group. *n* = 5 independent experiments; data were analysed by one‐way ANOVA. (c, d) CC‐930 (10 μM) or PD169316 (10 μM) alone did not increase apoptosis in U87 cells. Cells were pretreated with CC‐930 or PD169316 for 2 hr and then incubated with bepridil (25 μM) for 24 hr; only CC‐930 significantly suppressed bepridil‐induced apoptosis. *P < .05, apoptotic cell fraction in CC‐930 + bepridil group versus bepridil group. *n* = 5 independent experiments; data were analysed by one‐way ANOVA

We next investigated whether p38‐MAPK and JNK signalling were involved in bepridil‐induced cell death. A JNK inhibitor tanzisertib (CC‐930) and a p38‐MAPK inhibitor PD169316 were dissolved in DMSO to make stock solutions. Flow cytometry analysis shows that U87 cells treated with vehicle (0.1/% DMSO, vol/vol), CC‐930 (10 μM), or PD169316 (10 μM) had similar cell distribution in each phase of the cell cycle, while bepridil (25 μM) incurred G_0_/G_1_ cell cycle arrest (Figure 7a,b). This arresting effect was prevented by PD169316 but not CC‐930, indicating that the p38‐MAPK signalling is involved in bepridil‐induced cell cycle arrest. The Annexin V apoptosis assay demonstrates that CC‐930 and PD169316 did not activate apoptosis (Figure 7c,d). Bepridil (25 μM) increased the percentage of apoptotic cells after 24‐hr incubation. This effect was attenuated by CC‐930 but not PD169316, indicating that the JNK signalling is involved in bepridil‐induced apoptosis.

### 3.6 Functional expression of NCX isoforms and impact of bepridil on human astrocytes

**Figure 8:**
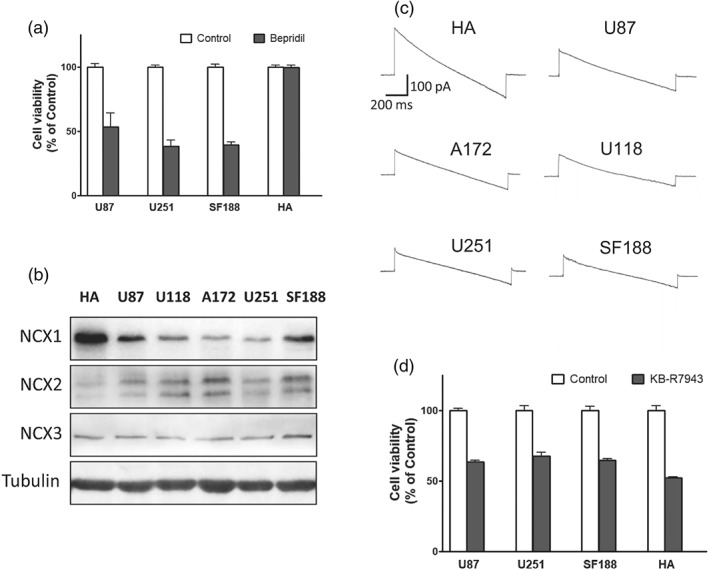
Impact of bepridil on human astrocytes (HA) and the Na^+^/Ca^2+^ exchanger (NCX) isoforms in each cell line. (a) Viability of U87, U251, and SF188 cells and HA after exposure to bepridil (25 μM, 48 hr); *n* = 5 independent tests in each group. (b) The bands of NCX1, NCX2, and NCX3 isoforms detected by Western blot analysis in the sample of HA and glioblastoma cell lines U87, U118, A172, U251, and SF188. (c) Representative recording of the NCX currents in HA and glioblastoma cell lines. (d) Viability of U87, U251, and SF188 cells and HA after exposure to KB‐R7943 (25 μM, 48 hr). *n* = 5 independent tests in each group

Glioblastoma arises from glial cells in the brain (Ostrom, Gittleman, Stetson, Virk, & Barnholtz‐Sloan, 2018). Bepridil was previously applied by Johnson & Johnson Pharmaceutical Research for the treatment of angina and hypertension. We here assessed viability of human astrocytes after exposed to bepridil (25 μM) for 48 hr and compared it with glioblastoma cells. Bepridil did not generate apparent cytotoxicity in human astrocytes, meanwhile markedly suppressed growth of U87, U251, and SF188 cells (Figure 8a). To explore why human astrocyte is not sensitive to NCX blocker bepridil, we measured expression of three NCX isoforms in astrocytes and glioblastoma cells. Western blot analysis shows that NCX1 expression is significantly higher in human astrocytes than glioblastoma cells (Figure 8b). But the level of NCX2 and NCX3 is not high; especially, NCX2 is even lower than that in glioblastoma cells. This observation is identical to the literature, documenting that in astrocytes, NCX1 is the most highly expressed among the three NCX isoforms (Boscia et al., 2016; Pappalardo, Samad, Black, & Waxman, 2014). We then decided to compare the activity of NCX among them by using whole‐cell patch‐clamp recording. The amplitude of the NCX currents in human astrocytes is much larger than glioblastoma cells (Figure 8c). After performing a calculation, we show current density of the NCX in human astrocytes is several‐fold higher than that in glioblastoma cells (Table S1). This high NCX activity and astrocyte's resistance to bepridil's toxicity most likely result from high expression of NCX1 isoform. On the other, KB‐R7943 is deleterious to astrocytes probably due to its multiple actions besides blocking NCX (Figure 8d).

### 3.7 Bepridil inhibits growth of brain‐grafted tumour cells in vivo

**Figure 9:**
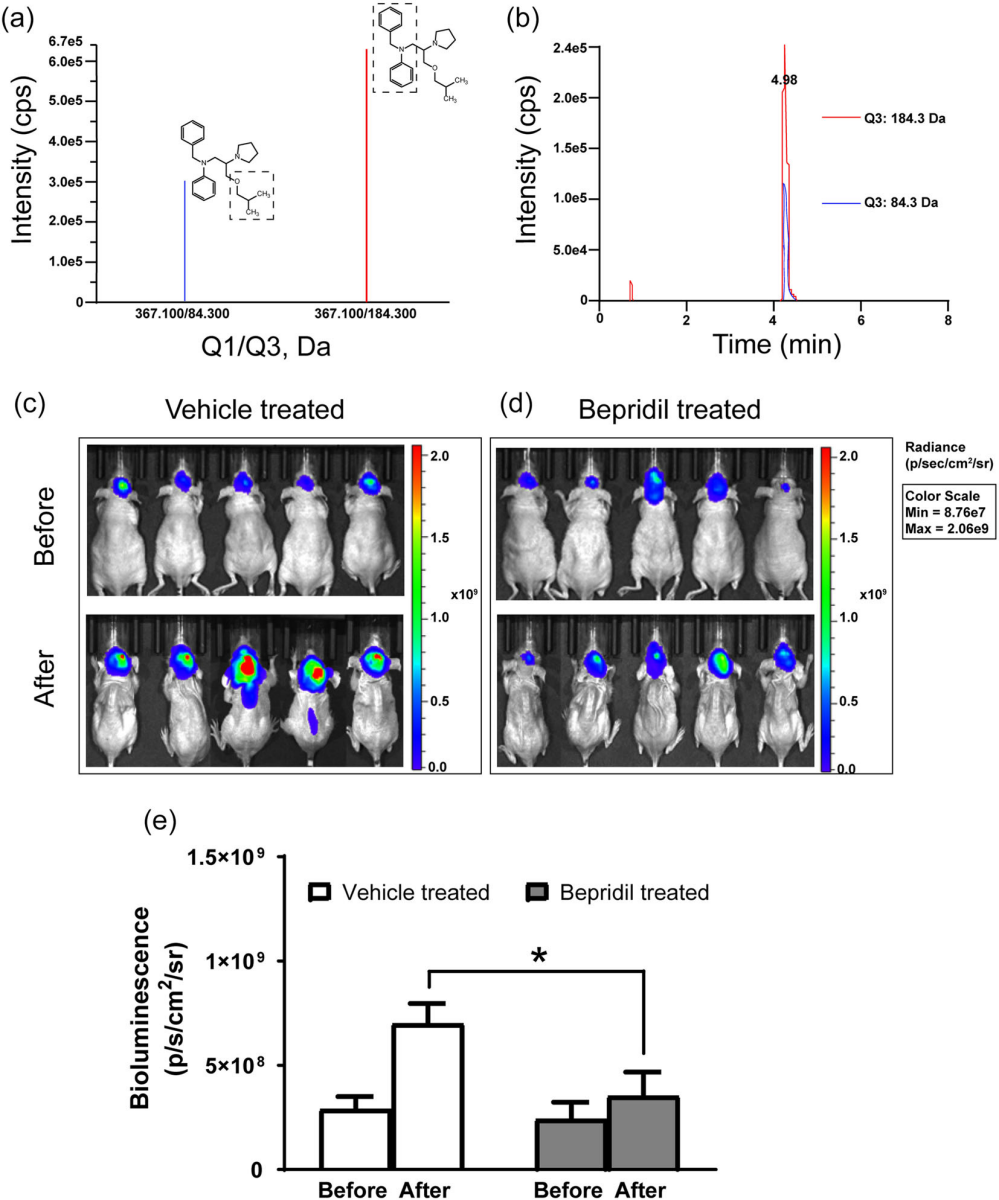
Bepridil crosses the BBB and inhibits growth of brain‐grafted glioblastoma. (a) Dotted box indicates the precursor to product ions (Q1 → Q3) selected for bepridil during quantitative optimization: 367.1 → 84.3 and 367.1 → 184.3 in positive ion mode. (b) Typical chromatogram of brain homogenate sample collected at 2 hr following systemic administration of bepridil (50 mg·kg^−1^, p.o.). (c, d) Bioluminescent images of U87‐luciferase glioblastoma cells implanted into the right cerebral striatum of nude mice. Images were taken by an IVIS Spectrum CT Imaging System before and after the period of treatment with bepridil or vehicle as described in methods. (e) Tumour size determined by bioluminescence and caliper measurement in each group before and after treatment. *P < .05, comparison between the two groups; *n* = 5 mice per group, analysed by Student's parametric unpaired *t* test

Bepridil has been shown to able to penetrate the blood–brain barrier (BBB) and reach the brain after oral administration or intraperitoneal injection at 50 mg·kg^−1^ body weight in two separate animal studies (Lipsanen et al., 2013; Mitterreiter et al., 2010). Our MS experiment shows that bepridil can cross the BBB and get into brain tissue (Figure 9a,b) after oral administration. We then tested the effect of bepridil on the growth of established glioblastoma U87‐Luc xenografts. U87‐Luc cells were stereotactically injected into the right striatum of male nude mice. Sixteen days after intracranial implantation, bepridil (50 mg·kg^−1^, p.o.) was administered once a day for 14 days. Control group received the same amount of vehicle treatment. Bioluminescent imaging of U87‐Luc cells in live mice brain was performed before treatment began and after treatment finished. Tumour size was determined through quantitative bioluminescence of live cells expressing luciferase under the IVIS Spectrum CT Imaging System. Vehicle treatment cannot hamper increasing tumour size, while bepridil successfully restrained tumour growth, yielding a significant volume difference between two groups (Figure 9c‐e). So, bepridil is also effective in suppressing growth of glioblastoma in vivo.

## 4 DISCUSSION AND CONCLUSION

The Ca^2+^ signalling in glioblastoma cells attracts increasing attention, and blockade of Ca^2+^ permeable channels was advised as a strategy to cure glioblastoma (Alptekin et al., 2015; Ding et al., 2010; Leclerc et al., 2016; Zhang et al., 2017). The present study proposes an alternative strategy other than blocking Ca^2+^ entry; this new approach is to block the forward NCX (Ca^2+^ exit mode) and cause Ca^2+^‐mediated injury in glioblastoma cells.

Bepridil and CB‐DMB are inhibitors that preferentially block the forward NCX; CB‐DMB is a more specific NCX blocker. SEA0400, SN‐6, and YM244769 only block the reverse NCX. We show that bepridil and CB‐DMB, at their concentration range for blocking the forward NCX, are toxic to glioblastoma cells. In contrast, SEA0400, SN‐6, and YM244769 produce very slight toxicity to glioblastoma cells. The cytotoxicity of bepridil and CB‐DMB to glioblastoma cells is likely from their blocking action on the forward NCX. In supporting this speculation, we found that both bepridil and CB‐DMB incur extracellular Ca^2+^ to flow into tumour cells and thus elevates [Ca^2+^]_i_. Bepridil or CB‐DMB‐caused Ca^2+^ influx results from blockade of the forward NCX. This blocking action occludes NCX‐mediated Ca^2+^ exit, but extracellular Ca^2+^ can enter a cell through other Ca^2+^‐permeable channels, therefore generating a net Ca^2+^ influx.

Elevation of [Ca^2+^]_i_ can modulate cell‐division cycle and cell death signalling (Humeau et al., 2018). We show that both bepridil and CB‐DMB can cause G_0_/G_1_ cell cycle arrest and apoptosis in glioblastoma cells; the Ca^2+^ chelator BAPTA‐AM attenuated bepridil or CB‐DMB‐induced apoptosis and cell cycle arrest. These data further proved that bepridil and CB‐DMB kill glioblastoma cells through blocking the forward NCX and increasing [Ca^2+^]_i_. KB‐R7943 incubation also induces G_0_/G_1_ arrest and apoptosis, but these cell death were not mediated by Ca^2+^ although KB‐R7943 also increases [Ca^2+^]_i_. KB‐R7943 initially was reported as an inhibitor of the reverse NCX; however, there is increasing evidence that KB‐R7943 also inhibits many other ionic channels (Barrientos, Bose, Feng, Padilla, & Pessah, 2009; Brustovetsky et al., 2011; Pezier, Bobkov, & Ache, 2009). In our study, KB‐R7943 is also toxic to normal astrocytes at the concentration range for it suppresses glioblastoma cells. We did not conduct further study to reveal intracellular signalling that mediates toxicity of KB‐R7943.

Our test of bepridil generates more encouraging results. Bepridil can penetrate the BBB and reach the brain. Animal experiment demonstrated that bepridil reduces in vivo growth of brain‐grafted glioblastoma. In addition, bepridil does not produce apparent cytotoxicity to human astrocytes when it markedly inhibits glioblastoma cells in vitro. This resistance to bepridil's cytotoxicity may result from high NCX1 expression and high NCX activity in astrocytes. The NCX1 expression is much lower in glioblastoma cells than in astrocytes. From the perspective of pharmacodynamics, the potency of a drug is proportional to the percentage of targets being occupied (Pugsley, Authier, & Curtis, 2008). When most NCX1s on glioblastoma cells are occupied by bepridil at an appropriate concentration, only a small fraction of NCX1s are blocked in astrocytes. Therefore, viability of astrocytes is not affected by bepridil when it already hurts glioblastoma cells.

The present study cannot completely exclude the possibility that other mechanisms also contribute to the sensitivity difference between astrocytes and glioblastoma cells. For example, cytosolic Na^+^ inactivates operation of the NCX1 and NCX3 (not NCX2), while increase of cytosolic Ca^2+^ activates all NCXs and also alleviates the Na^+^‐induced inactivation (Tal, Kozlovsky, Brisker, Giladi, & Khananshvili, 2016; Verkhratsky, Trebak, Perocchi, Khananshvili, & Sekler, 2018). In glioblastoma cells, the elevated [Ca^2+^]_i_ cannot activate NCXs because most NCXs were occupied and blocked by bepridil. In human astrocytes, NCX1 is the most highly expressed isoform, and only a small portion of NCX1s were blocked by bepridil. The remaining NCX1s can be readily activated by [Ca^2+^]_i_ and operate in the forward mode, which potentially underlies astrocyte's resistance to bepridil. Second, the reverse NCX operation accounts for [Ca^2+^]_i_ overload and ensuing injury in astrocytes when these glial cells are in resting, stimulating, or ischaemic conditions, especially when Na^+^‐driven uptake of neurotransmitters occurs (Reyes, Verkhratsky, & Parpura, 2012; Verkhratsky et al., 2018). It is possible that the reverse NCXs were blocked by bepridil in human astrocytes, and thereafter, the cytotoxicity was regressed, whereas this situation is different in glioblastoma and the cytotoxicity was augmented because the forward NCXs were inhibited.

The role of NCX has been well described in glial cells by these articles (Boscia et al., 2016; Parpura, Sekler, & Fern, 2016; Rose & Verkhratsky, 2016). But there are very few studies that report NCXs in the tissue of glioblastoma; particularly, it lacks data that compare NCXs between glioblastoma and glial cells. We here got a new observation that the NCX activity is higher in normal astrocytes than that in glioblastoma cells. The direction of NCX operation is controlled by change of membrane potential and gradients of Na^+^ and Ca^2+^, which constantly coordinate with neuronal activity. Conversely, the NCX operation tightly regulates Na^+^ and Ca^2+^ signalling, which manipulates neuronal excitability and neurotransmission. Down‐regulated NCX activity in glioblastoma could disrupt the crosstalk between glial and neuronal cells, leading to a detrimental ionic environment in the brain. So far, our materials verify our hypothesis and suggest that the plasma membrane NCX represents a new potential therapeutic target for glioblastoma.

Glioblastoma is the most common type of glioma that arises from glial cells in the brain or spinal cord. The annual incidence rate of glioblastoma in adults is between 0.6 and 3.7 per 100,000 persons (Ostrom et al., 2018). Patients with glioblastoma usually have a mean survival rate of 3–4 months if without treatment (Krex et al., 2007; Rao, 2003). Under combined treatment of radiotherapy and chemotherapy, the median survival time with grade IV glioblastoma can be prolonged to 15 months (Wen & Reardon, 2016). Currently, there is no cure for human glioblastoma. The present pharmacological study demonstrates that blocking the forward NCX causes [Ca^2+^]_i_ accumulation and Ca^2+^‐mediated demise of glioblastoma cells. Bepridil is effective in beating glioblastoma via blocking the forward NCX, thus may be used as a leading compound for developing new anti‐tumour drugs. Bepridil was previously approved by the Food and Drug Administration to treat angina but withdrawn later because it can induce new arrhythmias. We recommend that a structural modification is required to reduce the cardiac side effect of bepridil before it is re‐purposed to treat human glioblastoma.

## CONFLICT OF INTEREST

The authors declare no conflicts of interest.

## AUTHOR CONTRIBUTIONS

M.S. conceived this study, designed the experiments, and wrote this manuscript. H.‐J.H., S.‐S.W., Y.‐X.W., and Y.L. conducted the experiments and analysed the data. X.‐M.F., Y.S., and L.Z. contributed the essential reagents, tools, and techniques and contributed in method modification. H.‐Z.C. supervised this project.

## DECLARATION OF TRANSPARENCY AND SCIENTIFIC RIGOUR

This Declaration acknowledges that this paper adheres to the principles for transparent reporting and scientific rigour of preclinical research as stated in the *BJP* guidelines for https://bpspubs.onlinelibrary.wiley.com/doi/abs/10.1111/bph.14207, https://bpspubs.onlinelibrary.wiley.com/doi/full/10.1111/bph.14208, and https://bpspubs.onlinelibrary.wiley.com/doi/full/10.1111/bph.14206 and as recommended by funding agencies, publishers, and other organizations engaged with supporting research.

## Supporting information

Figure S1. Effect of the NCX blocker CB‐DMB on viability and the level of [Ca^2+^]_i_ of glioblastoma cells. (A) Viability of adult glioblastoma cell lines (U87, U251 and SF188) after exposed to the NCX blocker CB‐DMB at 0.1, 1.0, 2.0, 4.0 and 7.5 μM for 72 hours. *n* = 4 independent tests. (B) Flua‐4 AM loaded U87 cells were imaged and perfused with CB‐DMB (2 and 4 μM) in the solution containing 2 mM Ca^2+^. (C) In Ca^2+^ free solution, Ca^2+^ imaging before and after perfusion with CB‐DMB (4 μM), and then plus 5 μM thapsigargin to release Ca^2+^ from the endoplasmic reticulum. (D) U87 cells were pre‐treated with a Ca^2+^ chelator BAPTA‐AM (20 μM) for 2 hours, then imaged and perfused with CB‐DMB (4 μM). The imaging traces are representative of 3 separate experiments, and 12–15 cells were imaged per experiment.

Figure S2. Effect of the NCX blocker on the level of [Na^+^]_i_ of glioblastoma cells and detection of NCX isoforms in the cell nucleus. (A‐C) The Na^+^ imaging of U87 cells before and after perfusion with bepridil (50 μM), KB‐R7943 (50 μM) and CB‐DMB (4 μM). The imaging traces are representative of 3 separate experiments; 11–12 cells were imaged per experiment. (D) Detection of the NCX isoforms in the extracts of whole cells and nuclei of U87 by western blot analysis.

Figure S3. CB‐DMB caused cell cycle arrest and apoptosis in glioblastoma cells. (A‐B) Cell cycle assay of U87 cells incubated with CB‐DMB (3.5 μM) for 6, 12, 18 and 24 hours. CB‐DMB increased the proportion of cells in G_0_/G_1_ phase in a time‐dependent manner. (C) Cells were pretreated with BAPTA‐AM (10 μM) for 18 hours then incubated with CB‐DMB (3.5 μM) for 18 hours. BAPTA‐AM significantly attenuated CB‐DMB‐induced cell cycle arrest. *P < 0.05, G_0_/G_1_ phase of BAPTA‐AM + CB‐DMB vs. CB‐DMB alone; *n* = 5 independent tests. (D‐E) U87 cells were treated with CB‐DMB (3.5 μM) for 6, 12, 24 and 48 hours, then analyzed with Annexin V‐FITC/PI apoptosis assay. CB‐DMB incurred a time‐dependent increase of apoptotic cells (Annexin V^+^). (F) Cells were pretreated with BAPTA‐AM (10 μM) for 18 hours then incubated with CB‐DMB (3.5 μM) for 18 hours. BAPTA‐AM significantly suppressed CB‐DMB‐induced apoptosis. *n* = 5 independent tests; *P < 0.05, apoptotic cell fraction in BAPTA‐AM + CB‐DMB vs. CB‐DMB alone, with the parametric one‐way ANOVA followed by a Tukey's post‐hoc test.

Table S1. Density (mean ± SEM, pA pF^−1^) of trans‐membrane currents related to the NCX operation in human astrocytes (HA) and glioblastoma cells.
