# Supplementary figures and images for: Blockade of the forward Na+/Ca2+ exchanger suppresses the growth of glioblastoma cells through Ca2+‐mediated cell death
Source: Br J Pharmacol. 2019 Jun 17;176(15):2691–707. doi: 10.1111/bph.14692 (PMC6609550; doi:10.1111/bph.14692)

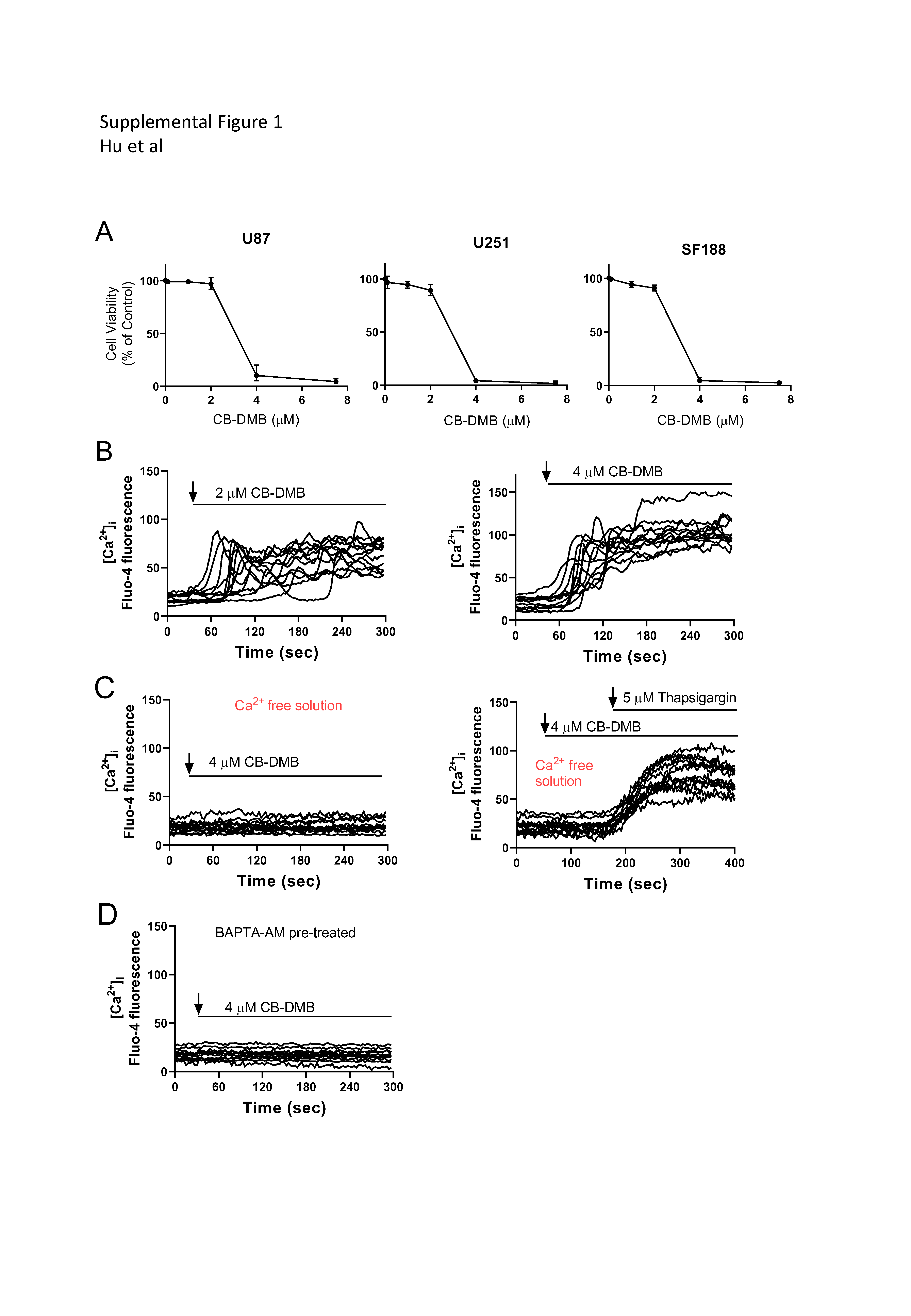

Supplement: Supplementary file 1 — Figure S1. Effect of the NCX blocker CB‐DMB on viability and the level of [Ca2+]i of glioblastoma cells. (A) Viability of adult glioblastoma cell lines (U87, U251 and SF188) after exposed to the NCX blocker CB‐DMB at 0.1, 1.0, 2.0, 4.0 and 7.5 μM for 72 hours. n = 4 independent tests. (B) Flua‐4 AM loaded U87 cells were imaged and perfused with CB‐DMB (2 and 4 μM) in the solution containing 2 mM Ca2+. (C) In Ca2+ free solution, Ca2+ imaging before and after perfusion with CB‐DMB (4 μM), and then plus 5 μM thapsigargin to release Ca2+ from the endoplasmic reticulum. (D) U87 cells were pre‐treated with a Ca2+ chelator BAPTA‐AM (20 μM) for 2 hours, then imaged and perfused with CB‐DMB (4 μM). The imaging traces are representative of 3 separate experiments, and 12–15 cells were imaged per experiment. [file BPH-176-2691-s001.tif]

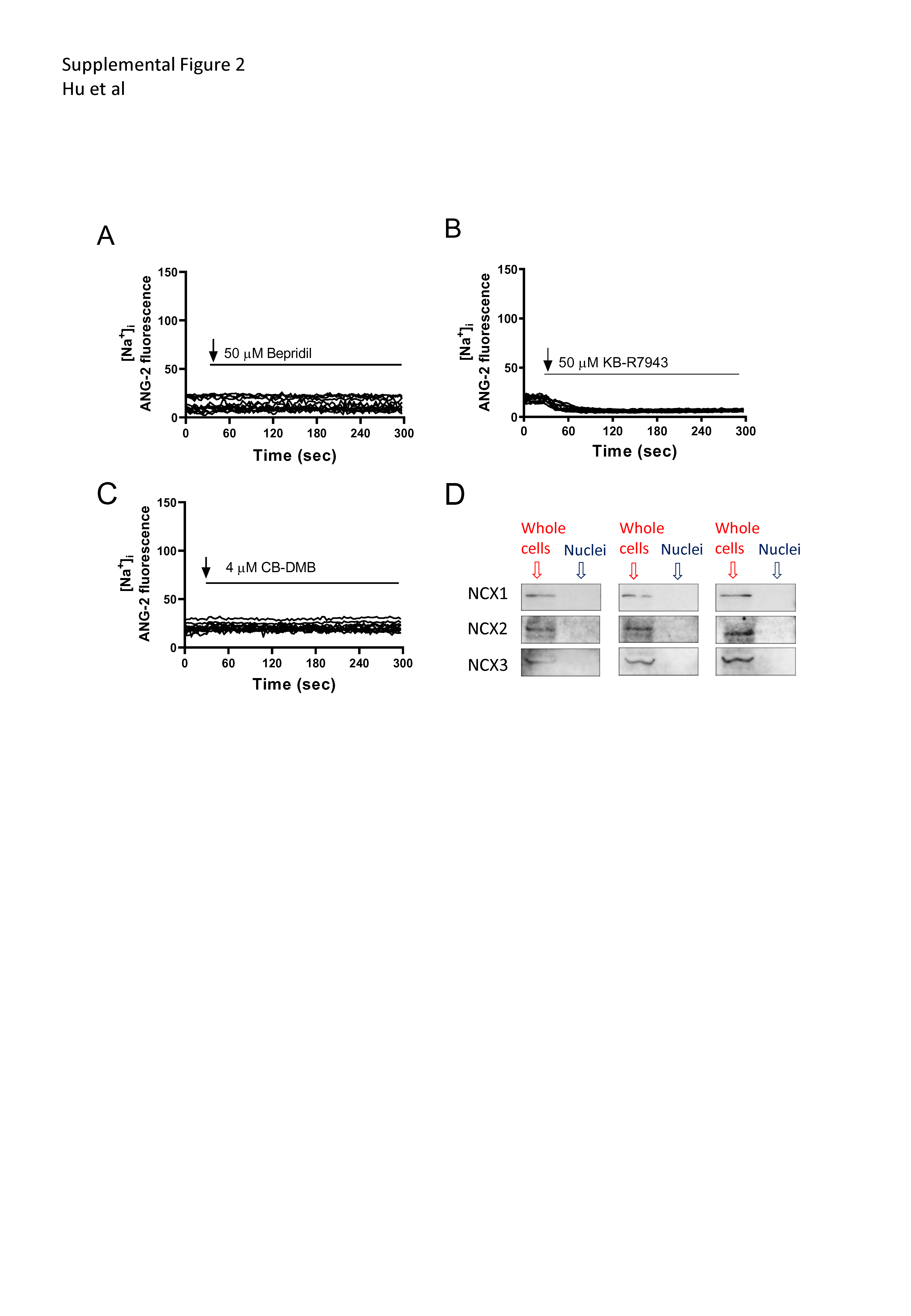

Supplement: Supplementary file 2 — Figure S2. Effect of the NCX blocker on the level of [Na+]i of glioblastoma cells and detection of NCX isoforms in the cell nucleus. (A‐C) The Na+ imaging of U87 cells before and after perfusion with bepridil (50 μM), KB‐R7943 (50 μM) and CB‐DMB (4 μM). The imaging traces are representative of 3 separate experiments; 11–12 cells were imaged per experiment. (D) Detection of the NCX isoforms in the extracts of whole cells and nuclei of U87 by western blot analysis. [file BPH-176-2691-s002.tif]

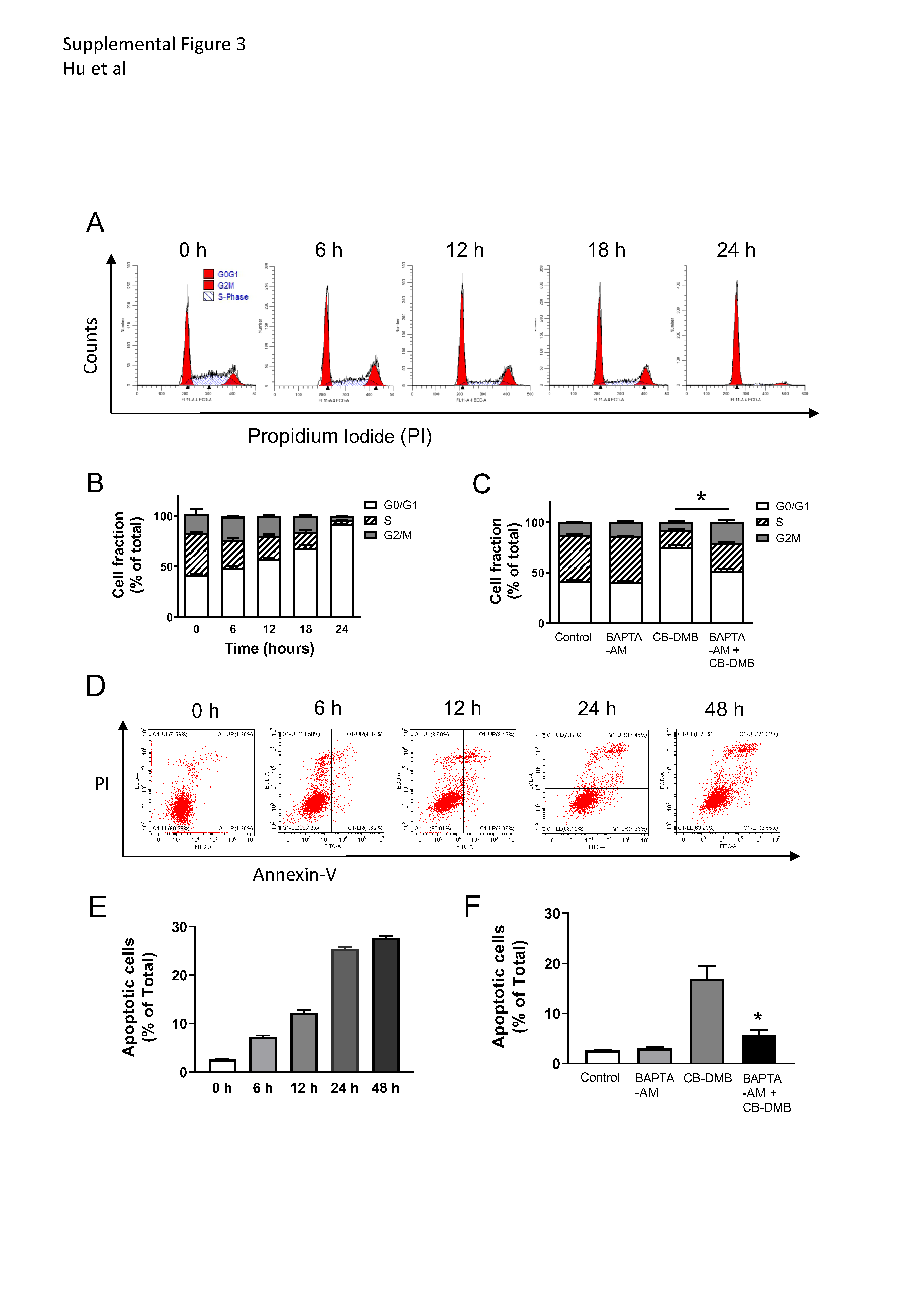

Supplement: Supplementary file 3 — Figure S3. CB‐DMB caused cell cycle arrest and apoptosis in glioblastoma cells. (A‐B) Cell cycle assay of U87 cells incubated with CB‐DMB (3.5 μM) for 6, 12, 18 and 24 hours. CB‐DMB increased the proportion of cells in G0/G1 phase in a time‐dependent manner. (C) Cells were pretreated with BAPTA‐AM (10 μM) for 18 hours then incubated with CB‐DMB (3.5 μM) for 18 hours. BAPTA‐AM significantly attenuated CB‐DMB‐induced cell cycle arrest. *P < 0.05, G0/G1 phase of BAPTA‐AM + CB‐DMB vs. CB‐DMB alone; n = 5 independent tests. (D‐E) U87 cells were treated with CB‐DMB (3.5 μM) for 6, 12, 24 and 48 hours, then analyzed with Annexin V‐FITC/PI apoptosis assay. CB‐DMB incurred a time‐dependent increase of apoptotic cells (Annexin V+). (F) Cells were pretreated with BAPTA‐AM (10 μM) for 18 hours then incubated with CB‐DMB (3.5 μM) for 18 hours. BAPTA‐AM significantly suppressed CB‐DMB‐induced apoptosis. n = 5 independent tests; *P < 0.05, apoptotic cell fraction in BAPTA‐AM + CB‐DMB vs. CB‐DMB alone, with the parametric one‐way ANOVA followed by a Tukey's post‐hoc test. [file BPH-176-2691-s003.tif]
