# Supplementary material for: Blockade of the forward Na+/Ca2+ exchanger suppresses the growth of glioblastoma cells through Ca2+‐mediated cell death
Source: Br J Pharmacol. 2019 Jun 17;176(15):2691–707. doi: 10.1111/bph.14692 (PMC6609550; doi:10.1111/bph.14692)
Supplement: Supplementary file 4 — Table S1. Density (mean ± SEM, pA pF−1) of trans‐membrane currents related to the NCX operation in human astrocytes (HA) and glioblastoma cells. [file BPH-176-2691-s004.docx]

Supplemental Table 1. Density (mean ± SEM, pA pF^-1^) of trans-membrane currents related to the NCX operation in human astrocytes (HA) and glioblastoma cells.

| Cell lines | Current density of  the reverse NCX | Current density of  the forward NCX | n |
| --- | --- | --- | --- |
| HA  U87  U118  A172  U251  SF188 | 13.9 ± 0.2  4.7 ± 0.2  2.3 ± 0.2  3.2 ± 0.2  2.1 ± 0.1  2.5 ± 0.1 | 5.7 ± 0.5  2.2 ± 0.3  1.2 ± 0.1  2.1 ± 0.1  1.4 ± 0.1  1.3 ± 0.1 | 5  3  3  3  3  3 |

Note: n is the number of independent recordings in each cell line.
